# Effects of Chemical Modifications on the Thermoresponsive Behavior of a PDMAEA-b-PNIPAM-b-POEGA Triblock Terpolymer

**DOI:** 10.3390/polym12061382

**Published:** 2020-06-19

**Authors:** Despoina Giaouzi, Stergios Pispas

**Affiliations:** Theoretical and Physical Chemistry Institute, National Hellenic Research Foundation, 48 Vassileos Constantinou Avenue, 11635 Athens, Greece; dgiaouzi@gmail.com

**Keywords:** triblock terpolymer, thermoresponsive, RAFT polymerization, chemical modification, stimuli responsive, self-assembly

## Abstract

In this work, the synthesis, selective chemical modifications, and self-assembly behavior in aqueous media of a novel poly(2-(dimethylamino)ethyl acrylate)_20_-b-poly(N-isopropylacrylamide)_11_-b-poly(oligo ethylene glycol methyl ether acrylate)_18_ (PDMAEA_20_-b-PNIPAM_11_-b-POEGA_18_) dual-responsive (pH and temperature) and triply hydrophilic amino-based triblock terpolymer are reported. The amine functional triblock terpolymer was synthesized by sequential reversible addition fragmentation chain transfer polymerization (RAFT) polymerization and molecularly characterized by size exclusion chromatography (SEC) and ^1^H-NMR spectroscopy that evidenced the success of the three-step polymerization scheme. The tertiary amine pendant groups of the PDMAEA block were chemically modified in order to produce the Q_1_PDMAEA_20_-b-PNIPAM_11_-b-POEGA_18_ as well as the Q_6_PDMAEA_20_-b-PNIPAM_11_-b-POEGA_18_ quaternized triblock terpolymers (Q_1_ and Q_6_ prefixes show the number of carbon atoms (C1 and C6) attached on the PDMAEA groups) using methyl iodide (CH_3_I) and 1-iodohexane (C_6_H_13_I) as the quaternizing agents and the SPDMAEA_20_-b-PNIPAM_11_-b-POEGA_18_ sulfobetainized triblock terpolymer using 1,3 propanesultone (C_3_H_6_O_3_S) as the sulfobetainization agent. The self-assembly properties of the triblock terpolymers in aqueous solutions upon varying temperature and solution pH were studied by light scattering and fluorescence spectroscopy experiments. The novel triblock terpolymers self-assemble into nanosized aggregates upon solution temperature rise above the nominal lower critical solution temperature (LCST) of the temperature-responsive PNIPAM block. The remarkable stimuli-responsive self-assembly behavior of the novel triblock terpolymers in aqueous media make them interesting candidates for biomedical applications in the fields of drug and gene delivery.

## 1. Introduction

Diblock copolymers have garnered the ever-increasing scientific interest over the past decades regarding the design and synthesis of novel copolymer systems by implementing facile polymerization techniques as well as the in-depth comprehension of behavioral motifs intending to their utilization in a wide range of applications [1,2,3,4,5]. Controlled/living polymerization procedures of all types have been followed for the synthesis of diblock copolymers of the AB type (A and B represent the different blocks) [6,7], which have been researched extensively as synthetic materials for various applications in the fields of nanotechnology [8]. Nevertheless, the addition of one more stimuli-responsive block to the AB type diblock copolymers can provide novel linear stimuli-responsive triblock terpolymers of the ABC type with ameliorated functionality and structural complexity at the molecular and supramolecular level in comparison to the diblock copolymer precursors [9,10]. Controlled radical polymerization methodologies such as nitroxide-mediated radical polymerization (NMP) [11], atom transfer radical polymerization (ATRP) [12,13] and reversible addition fragmentation chain transfer polymerization (RAFT) [14,15] have been utilized for the synthesis of linear triblock terpolymers. However, RAFT polymerization demonstrates some significant advantages, which include the synthesis of well-defined polymers with controlled molecular characteristics and complex architectures, under less strict experimental conditions, the compatibility with a wide variety of functional and biocompatible monomers, as well as the absence of non-biocompatible inorganic catalysts [16,17,18,19,20]. Based on the above-mentioned features, RAFT polymerization has been used widely for the synthesis of several stimuli-responsive triblock terpolymers [21,22,23,24] that can be utilized for advanced biomedical applications, such as drug and/or gene delivery [25,26].

Stimuli-responsive polymers are a class of synthetic materials that have attracted considerable interest recently because of their unique ability to form self-assembled nanostructures in aqueous media in response to a range of physicochemical stimuli, demonstrating a wide range of supramolecular morphologies such as spherical or worm-like micelles and vesicles [27,28]. In particular, stimuli-responsive triblock terpolymers of the ABC type containing two different stimuli-responsive blocks and a third one that is a non-responsive hydrophobic or hydrophilic block have gained increased interest on account of their unique macromolecular architecture and fascinating aqueous solution properties, as well as of their potential applications in the fields of nanomedicine [29,30,31,32,33,34]. The amphiphilicity of stimuli-responsive triply hydrophilic triblock terpolymers, and thus their capability of self-assembling in aqueous media, can be manipulated after being exposed to one or more external stimuli such as temperature [35], pH [36,37,38], and ionic strength [39]. Thermo- and pH-responsive polymers are two of the most significant classes of stimuli-responsive polymers owing to their valuable applications as nanosized carriers for the triggered and targeted delivery of therapeutic molecules, including hydrophobic drugs or genes, in the human body [40,41,42,43]. Thermoresponsive polymers are classified in two main subcategories based on their temperature-dependent solubility properties in aqueous media. The first subcategory includes polymers that become dehydrated/hydrophobic upon heating over a lower critical solution temperature (LCST) and the second one includes polymers that become hydrated/hydrophilic upon heating over a UCST value (upper critical solution temperature value) [44,45]. Among various thermoresponsive polymers, PNIPAM (poly(N-isopropylacrylamide))-based polymers have received great interest due to its phase transition temperature being very close to human body temperature (LCST of 32 °C) and thus render it a prominent candidate for biomedical applications [46,47,48,49,50,51,52,53]. Moreover, pH-responsive polymers are of outstanding significance owing to their cationic nature and their pH swelling/deswelling properties in aqueous media, which are properties that make them valuable candidates in the field of gene therapy [54,55,56,57,58,59]. The poly(2-(dimethylamino)ethyl acrylate-based (PDMAEA) polymers, the acrylate version of poly(2-(dimethylamino)ethyl methacrylate (PDMAEMA), respond to pH variations since PDMAEA presents a pKa = 8.41) [60], and they are gaining extensive scientific attention because of their potential utilization as non-viral synthetic carriers for gene delivery. The PDMAEA polymer exhibits several advantages including low cytotoxicity in cultured cells, pH-independent self-catalyzed degradation in aqueous environment, sufficient loading capacity, as well as an absence of immunogenicity, which are features that emerge it an alternative carrier to viral gene delivery systems for nucleic acids or proteins delivery [60,61,62]. 

In the current work, the synthesis of a novel doubly responsive poly(2-(dimethylamino)ethyl acrylate)-b-poly(N-isopropylacrylamide)-b-poly(oligo ethylene glycol methyl ether acrylate) (PDMAEA-b-PNIPAM-b-POEGA) triblock terpolymer by utilizing sequential three-step RAFT polymerization methodologies is presented. In addition, the chemical modifications of the tertiary amine groups of the PDMAEA block, as well as the stimuli-responsive self-assembly behavior of all the obtained triblock terpolymers, are studied in aqueous media. The selection of these kinds of monomers in the same triblock terpolymer was made due to the stimuli-responsive properties of the PNIPAM and PDMAEA blocks, as well as the high hydrophilicity and biocompatible character of the POEGA block. The obtained triblock terpolymer was molecularly characterized by SEC and ^1^H-NMR spectroscopy in order to confirm the success of the three-step synthetic scheme. Post-polymerization chemical modification reactions of the tertiary side groups of PDMAEA block were accomplished by using methyl iodide (CH_3_I) and 1-iodohexane (C_6_H_13_) as the quaternizing agents and 1,3-propanesultone (C_3_H_6_O_3_S) as the sulfobetainization agent. In this way, the PDMAEA block transformed into a strong cationic polyelectrolyte, resulting in novel Q_1_PDMAEA-b-PNIPAM-b-POEGA and Q_6_PDMAEA-b-PNIPAM-b-POEGA triblock terpolymers, with increased hydrophobicity in the latter case, and a block with zwitterionic character in the SPDMAEA-b-PNIPAM-b-POEGA sulfobetainized triblock terpolymer. In this study, the synthesis of a triple hydrophilic triblock terpolymer consisting of two stimuli-responsive blocks (PDMAEA and PNIPAM blocks) and one invariable hydrophilic block (POEGA), as well as the chemical modifications of the amine PDMAEA block in the amino-based triblock terpolymer are reported for the first time. Furthermore, the chemical modifications of the tertiary amine side groups of PDMAEA-based polymers have obtained rather limited scientific attention compared to the PDMAEMA-based polymers, in which the quaternization reactions have been studied extensively. In the present systems, the self-assembly properties of all obtained triblock terpolymers can be manipulated by varying the solution conditions such pH (pH-dependent PDMAEA block) and temperature (temperature-dependent PNIPAM block) in the case of the amino-based triblock terpolymer and by changing the solution temperature in the cases of chemically modified triblock terpolymers, taking into consideration also their strong polyelectrolyte properties. The triblock terpolymers (amine and chemically modified) self-assembling properties in aqueous media were investigated by conducting light scattering and fluorescence spectroscopy measurements. As the temperature rises above the LCST value of PNIPAM block, all obtained triblock terpolymers display thermoresponsive character and thus self-assemble into nanostructures in aqueous media even though they possess a rather low amount of the thermoresponsive PNIPAM block, in comparison with other two constituting blocks.

## 2. Materials and Methods

### 2.1. Materials 

The used monomers, N-isopropyl acrylamide (NIPAM, ≥99%), 2-(dimethylamino) ethyl acrylate (DMAEA, 98%) and poly(oligo ethylene glycol methyl ether acrylate) (OEGA, average M_n_ = 480 g/mol) were obtained from Sigma-Aldrich. The purification of the monomers was achieved as follows: NIPAM monomer was recrystallized from n-hexane/benzene (4:1), while DMAEA and OEGA monomers were passed through an inhibitor-removing column. 1,4-Dioxane (99.8%), acquired from Sigma-Aldrich, was placed over molecular sieves before use in order to remove moisture. 2,2 Azobisisobutyronitrile (AIBN) was recrystallized from ethanol. 2-(Dodecylthiocarbonothioylthio)-2-methylpropionionic acid (DDMAT), Tetrahydrofuran (THF, 99.9%), methyl iodide (CH_3_I), 1-iodohexane (C_6_H_13_I), 1,3-propanesultone (C_3_H_6_O_3_S), pyrene, and other reagents were purchased from Sigma-Aldrich and utilized as received without further purification.

### 2.2. Synthesis of PDMAEA Homopolymer

The PDMAEA homopolymer was synthesized via RAFT polymerization utilizing 2-(dodecyltiocarbonothioylthio)-2-methylpropionionic acid (DDMAT) as the chain transfer agent (CTA). DMAEA (2 g), DDMAT (0.1458 g, 0.4 mmol), AIBN (0.0064 g, 0.04 as a solution in 1,4-dioxane) (moles DDMAT: moles AIBN = 10:1) were dissolved in 10 mL of 1,4-dioxane, and the solution was placed in a round-bottom flask (25 mL) equipped with a magnetic stirrer and fitted with a rubber septum. Then, the solution was degassed by passing through pure N_2_ gas bubbling for 15 min and immersed in a thermostated oil bath at 65 °C for 7 h. The polymerization reaction was terminated by placing the flask in a refrigerator (−20 °C) for 10 min and by subsequent exposure to air. The PDMAEA homopolymer was isolated by precipitation in excess of n-hexane, and the yellow product was dried for 48 h in a vacuum oven at ambient temperature. After that period, the PDMAEA macro-CTA (yield: 60%) was collected before being utilized for the polymerization of NIPAM monomer. Size exclusion chromatography (SEC) measurement displays that the weight average molecular weight (M_w_) is 2800 g mol^−1^ and the molecular weight dispersity index (M_w_/M_n_) is 1.17.

### 2.3. Synthesis of PDMAEA-b-PNIPAM Block Copolymer

The synthetic procedure of the intermediate PDMAEA-b-PNIPAM block copolymer is described in the following: PDMAEA macro-CTA (0.7 g, 0.259 mmol), NIPAM (0.3 g, 2.65 mmol), and AIBN (0.00426 g, 0.0259 mmol) were dissolved in 5 mL of 1,4-dioxane. The polymerization solution was placed in a 25 mL round-bottom flask with a magnetic stirrer and fitted with a septum. After 15 min degassing by pure N_2_ gas bubbling, the flask was immersed in a thermostated oil bath at 70 °C for 6 h. After the termination of the reaction, by freezing the polymerization solution at –20 °C and by exposure to air, the PDMAEA-b-PNIPAM block copolymer was isolated by precipitation in excess of n-hexane. After drying in a vacuum oven for 48 h, the block copolymer was collected (yield: 97%) and stored before being utilized as macro-CTA for the polymerization of OEGA monomer. The weight average molecular weight (M_w_) determined by SEC is 4100 g mol^−1^ and the molecular weight dispersity index (M_w_/M_n_) is 1.28. 

### 2.4. Synthesis of PDMAEA-b-PNIPAM-b-POEGA Triblock Terpolymer

The synthetic route for the preparation of the PDMAEA-b-PNIPAM-b-POEGA triblock terpolymer is described in the following: the PDMAEA-b-PNIPAM diblock copolymer was used as the macro-CTA, AIBN as the radical initiator, and 1,4-dioxane as the polymerization solvent. PDMAEA-b-PNIPAM (0.3 g, 0.0779 mmol), OEGA (0.7 g, 1.458 mmol), AIBN (0.002558 g, 0.01558 mmol), and 5 mL of 1.4-dioxane were added in a 25 mL round-bottom flask equipped with a magnetic stirrer and a rubber septum. Then, the solution was degassed by passing through pure nitrogen gas bubbling for 15 min, and the round bottom flask was put in an oil bath at 70 °C for 24 h. Subsequently, the polymerization reaction was quenched by cooling the solution in refrigerator at −20 °C and by exposure to air. The triblock terpolymer was isolated by precipitation in excess of n-hexane and dried in a vacuum oven for 48 h. The dry PDMAEA-b-PNIPAM-b-POEGA was collected (yield: 68%) and stored in a fridge for further utilization. The weight average molecular weight (M_w_) is 12,800 g mol^−1^ and the molecular weight dispersity index (M_w_/M_n_) is 1.57; both values were determined by size exclusion chromatography (SEC).

### 2.5. Chemical Modifications of Triblock Terpolymer

#### 2.5.1. Synthesis of Q_1_PDMAEA-b-PNIPAM-b-POEGA Triblock Terpolymer

The pH-dependent PDMAEA block of the triblock terpolymer was quaternized by using iodomethane (CH_3_I) so as to produce the Q_1_PDMAEA-b-PNIPAM-b-POEGA triblock terpolymer. The quaternization reaction procedure for the terpolymer is as follows: in a round-bottom flask, the PDMAEA-b-PNIPAM-b-POEGA terpolymer was dissolved in THF solvent (2% *w*/*v*) and subsequently an excess of CH_3_I quaternizing agent was added to the solution (moles CH_3_I: moles amine = 2). The reaction was left for 24 h under stirring at room temperature. After this period of time, the solvent was evaporated using a rotary evaporator and the almost dry product was collected and placed in a vacuum oven for 24 h in order to ensure complete drying of the material.

#### 2.5.2. Synthesis of Q_6_PDMAEA-b-PNIPAM-b-POEGA Triblock Terpolymer

The PDMAEA block of the triblock terpolymer was modified by 1-iodohexane (C_6_H_13_I) to yield the quaternized Q_6_PDMAEA-b-PNIPAM-b-POEGA triblock terpolymer. The quaternization reaction is described in the following: the triblock terpolymer was dissolved in THF (2% *w*/*v*) in a round-bottom flask, an excess of 1-iodohexane was added to the polymer solution (moles CH_3_I: moles amine = 2), and the quaternization reaction was left under stirring for 72 h at room temperature. After that period, a rotary evaporator was utilized in order to remove the solvent and subsequently the hydrophobically modified quaternized triblock terpolymer was collected and dried further in a vacuum oven for 24 h. 

#### 2.5.3. Synthesis of SPDMAEA-b-PNIPAM-b-POEGA Triblock Terpolymer

The PDMAEA block of triblock terpolymer was sulfobetainized utilizing 1,3-propanesultone (C_3_H_6_O_3_S) in order to give an SPDMAEA-b-PNIPAM-b-POEGA zwitterionic triblock terpolymer. The functionalization procedure is described in the following: in a round-bottom flask, the triblock terpolymer was dissolved in THF (2% *w*/*v*), and then 1,3-propanesultone as the sulfobetainizing agent (moles C_3_H_6_O_3_S/moles amine = 1.5) was added to the solution. The reaction was allowed to stir for 72 h at room temperature. After this period, the solvent was evaporated with a rotary evaporator, and the sulfobetainized triblock terpolymer was dried in a vacuum oven for 24 h. 

### 2.6. Self-Assembly of Triblock Terpolymer and Its Derivatives in Aqueous Solutions

The stock solutions of triblock terpolymer and its derivatives were prepared as follows: a weighted quantity of terpolymer was dissolved directly in the appropriate volume of distilled water. The solutions were allowed overnight at ambient temperature in order to ensure the complete dissolution of the triblock terpolymers. The PDMAEA-b-PNIPAM-b-POEGA triblock terpolymer stock solutions were prepared at three different pHs—3, 7, and 10—and the sample was measured fresh in order to avoid self-catalyzed hydrolysis of the PDMAEA block. The solution pH was regulated by the addition of proper amounts of 0.1 M solutions of HCl or NaOH. The concentration of all solutions measured was 1 × 10^−3^ g/mL. 

### 2.7. Characterization Methods

Size exclusion chromatography (SEC) was utilized in order to determine the molecular weights and the molecular weight distributions of the synthesized polymers. The SEC system was equipped with a Waters 1515 isocratic pump, a set of three μ-Styragel mixed bed columns (porosity size from 10^2^ to 10^6^Å), a Waters 2414 refractive index detector (at 40 °C), and controlled via Breeze software. Tetrahydrofuran (THF) solvent (containing 5% *v*/*v* triethylamine) was the mobile phase, at a flow rate of 1 ml/min and at 30 °C. Linear polystyrene standards with narrow molecular weight distributions and average molecular weights in the range of 1200–152,000 g/mol were used for calibration of the set-up. The concentrations of all the polymers analyzed were in the range of 2–4 mg/mL. 

^1^H-NMR spectra of the triblock terpolymers in deuterated solvents (CDCl_3_ and D_2_O) were recorded on a Varian 300MHz spectrometer and analyzed by Vjnmr software. Tetramethylsilane (TMS) was utilized as the internal standard, and the chemical shifts are reported in ppm.

PDMAEA-b-PNIPAM-b-POEGA: ^1^H-NMR (CDCl_3_, ppm): 3.63(*36H*, -(C*H_2_*C*H_2_*O)*_9_*, 3.36 (*3H*, -(CH_2_CH_2_O)_9_C*H_3_*-), 2.53 (*6H,* -N(C*H_3_*)*_2_*, 1.13 (*6H*, -NHCH(C*H_3_*)*_2_*.

Q_1_PDMAEA-b-PNIPAM-b-POEGA: ^1^H-NMR (D_2_O, ppm): 3.63(*36H*, -(C*H_2_*C*H_2_*O)*_9_*, 3.36 (*3H*, -(CH_2_CH_2_O)_9_C*H_3_*-), 1.13 (*6H*, -NHCH(C*H_3_*)*_2_*.

Q_6_PDMAEA-b-PNIPAM-b-POEGA: ^1^H-NMR (D_2_O, ppm): 3.63(36H, -(C*H_2_*C*H_2_*O)*_9_,* 3.36 (*3H*, -(CH_2_CH_2_O)_9_C*H_3_*-), 1.13 (*6H*, -NHCH(C*H_3_*)*_2_*

SPDMAEA-b-PNIPAM-b-POEGA: ^1^H-NMR (D_2_O, ppm): 3.63(*36H*, -(C*H_2_*C*H_2_*O)*_9_*, 3.36 (*3H*, -(CH_2_CH_2_O)_9_C*H_3_*-), 1.13 (*6H*, -NHCH(C*H_3_*).

Dynamic light scattering studies, in the angular range 30° to 150°, were carried out on an AVL/CGS-3 compact goniometer instrument (AVL GmbH, Germany), equipped with an ALV/LSE-5003 light scattering electronics unit for stepper motor drive and limit switch control, as well as an ALV-5000/EPP multi-tau digital correlator with 288 channels. The light source was a JDS Uniphase 22 mW He-Ne laser with a wavelength of 632.8 nm. Moreover, the temperature control of the measurement cell was achieved through a Polyscience 9102A12E water bath circulator. The autocorrelation functions were analyzed using the cumulants method and the CONTIN software. All solutions were filtered carefully through hydrophilic PVDF (polyvinyl difluoride) syringe filters (Membrane Solutions) in order to eliminate dust particles before measurements. Thereafter, the polymer solutions were transfered into standard cylindrical quartz cuvettes and left to equilibrate for 15 min. For temperature-dependent measurements, the polymer solutions were measured in the range from 25 to 55 °C in steps of 5 °C. Furthermore, the amine triblock terpolymer was studied at different solution pHs by directly dissolving the polymer in aqueous solution of three different pHs (pH = 3, 7, and 10). Before the dissolution of triblock terpolymers, the pH adjustment was achieved by the addition of 0.1 M HCl or NaOH solutions in distilled water. Static light scattering (SLS) measurements were performed on the same instrument and at the same temperature and angular range as in dynamic light scattering (DLS) measurements. Toluene was the calibration standard. The SLS results were determined using Zimm plots. The concentration for all samples was 1 × 10^−3^ g mL^−1^.

Electrophoretic light scattering measurements were carried out using a Nano Zeta Sizer (Malvern Instrument Ltd., Malvern, UK) equipped with a 4 mW solid-state laser at a wavelength of 633 nm and at 173° fixed backscattering angle. The ζ-potential values were obtained as the average of 50 runs, and the Henry correction of Smoluchowski equation was used to extract the data, after equilibration of the polymer solutions at 25 °C and 45 °C, respectively. 

Fluorescence spectroscopy experiments were conducted on a Fluorolog-3 Jobin Yvon-Spex spectrofluometer (model GL3-21) so as to examine the internal polarity of triblock terpolymers (amine and quaternized) in aqueous media at 25 °C and 45 °C, respectively, using pyrene as the fluorescence probe. The excitation wavelength utilized for the experiments was 335 nm, and the obtained emission spectra were recorded in the region 355–500 nm. The preparation of stock solutions for the fluorescence spectroscopy (FS) experiments is described in the following: 1 μL of pyrene stock solution (1 mM) in acetone was transfered into the vial containing 1 mL of aqueous terpolymer solution using a microsyringe. The solutions were allowed for acetone to evaporate overnight before being measured. Excimer formation was not observed for any of the triblock terpolymer solutions studied.

## 3. Results and Discussion

### 3.1. PDMAEA-b-PNIPAM-b-POEGA Triblock Terpolymer Synthesis, Chemical Modifications and Molecular Characterization

**Scheme 1:**
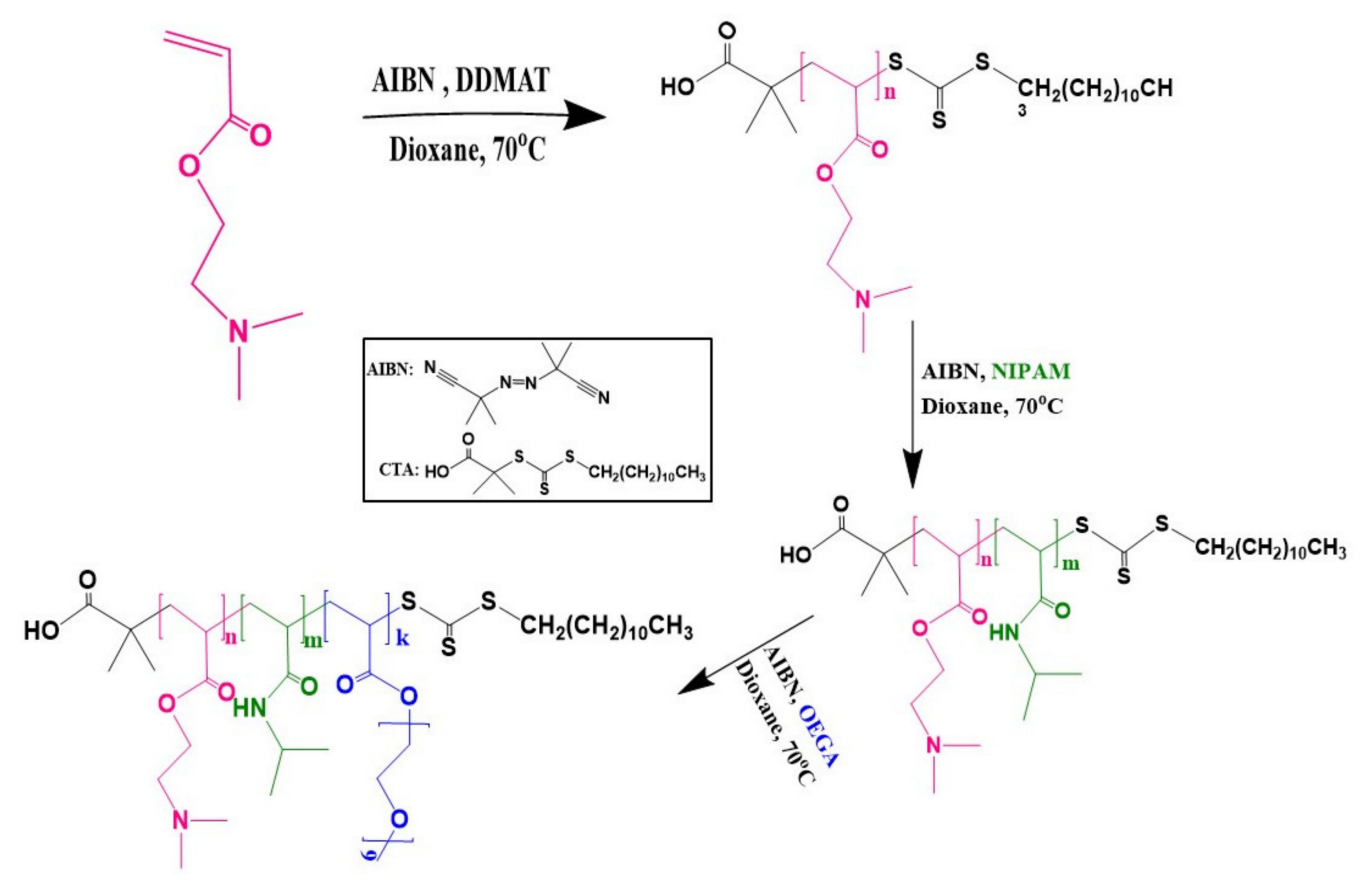
Synthesis route for poly(2-(dimethylamino)ethyl acrylate)_20_-b-poly(N-isopropylacrylamide)_11_-b-poly(oligo ethylene glycol methyl ether acrylate)_18_ (PDMAEA-b-PNIPAM-b-POEGA) triblock terpolymer preparation via sequential reversible addition fragmentation chain transfer polymerization (RAFT) polymerization.

The doubly stimuli-responsive PDMAEA_20_-b-PNIPAM_11_-b-POEGA_18_ triblock terpolymer (where the subscripts state the monomeric units in each block of the triblock terpolymer) was successfully synthesized via a sequential RAFT polymerization procedure, as depicted in Scheme 1.

The PDMAEA homopolymer and PDMAEA-b-PNIPAM diblock copolymer were used as the precursors for the synthesis of the PDMAEA_20_-b-PNIPAM_11_-b-POEGA_18_ triblock terpolymer. The chosen CTA was the DDMAT, because it is known from other studies [63,64] that it is reactive for these kinds of monomers, resulting in well-defined polymers with controlled molecular weights, as well as rather narrow and symmetrical molecular weight distributions.

**Figure 1:**
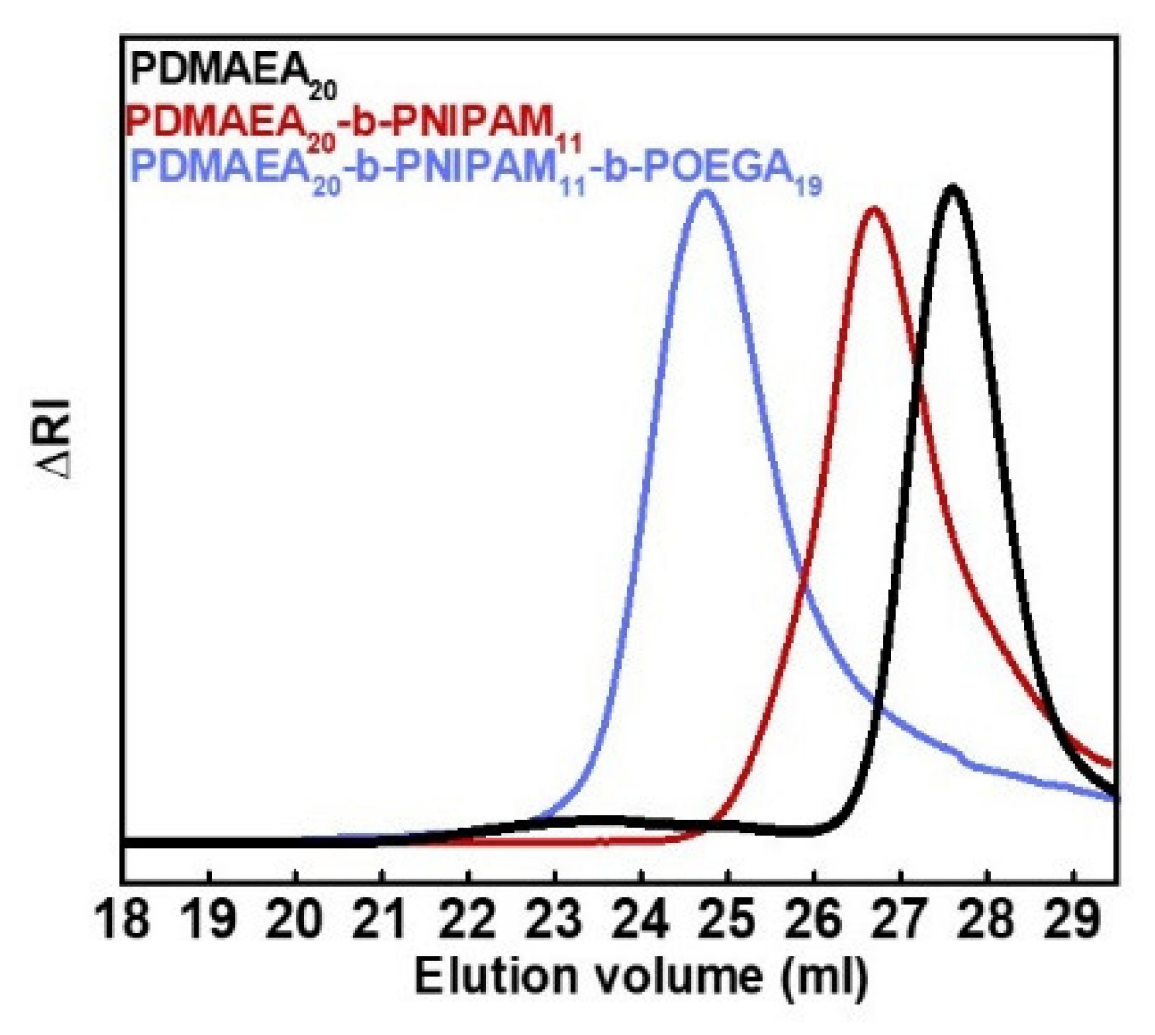
Size exclusion chromatography (SEC) traces of PDMAEA_20_ homopolymer, PDMAEA_20_-b-PNIPAM_11_ diblock copolymer, and PDMAEA_20_-b-PNIPAM_11_-POEGA_18_ triblock terpolymer in tetrahydrofuran (THF, containing 5% Et_3_N).

SEC measurements were conducted after each polymerization step in order to indicate the successful formation of each resulting block. It can be observed that after the polymerization of each monomer, the molecular distributions shift to higher molecular weights (Figure 1). SEC chromatographs confirmed the success of the above depicted synthetic route and revealed that the three-step RAFT polymerization methodology followed resulted in a well-defined PDMAEA_20_-b-PNIPAM_11_-POEGA_18_ triblock terpolymer with narrow and almost symmetrical weight distribution with a small amount of tail, indicating an almost integrated reinitiation of each polymerization step and definite extension of the sequential macromolecular chain generated. After three sequential polymerization steps, the molecular weight polydispersities are within the range ordinarily mentioned for RAFT polymerization methodologies. 

**Scheme 2:**
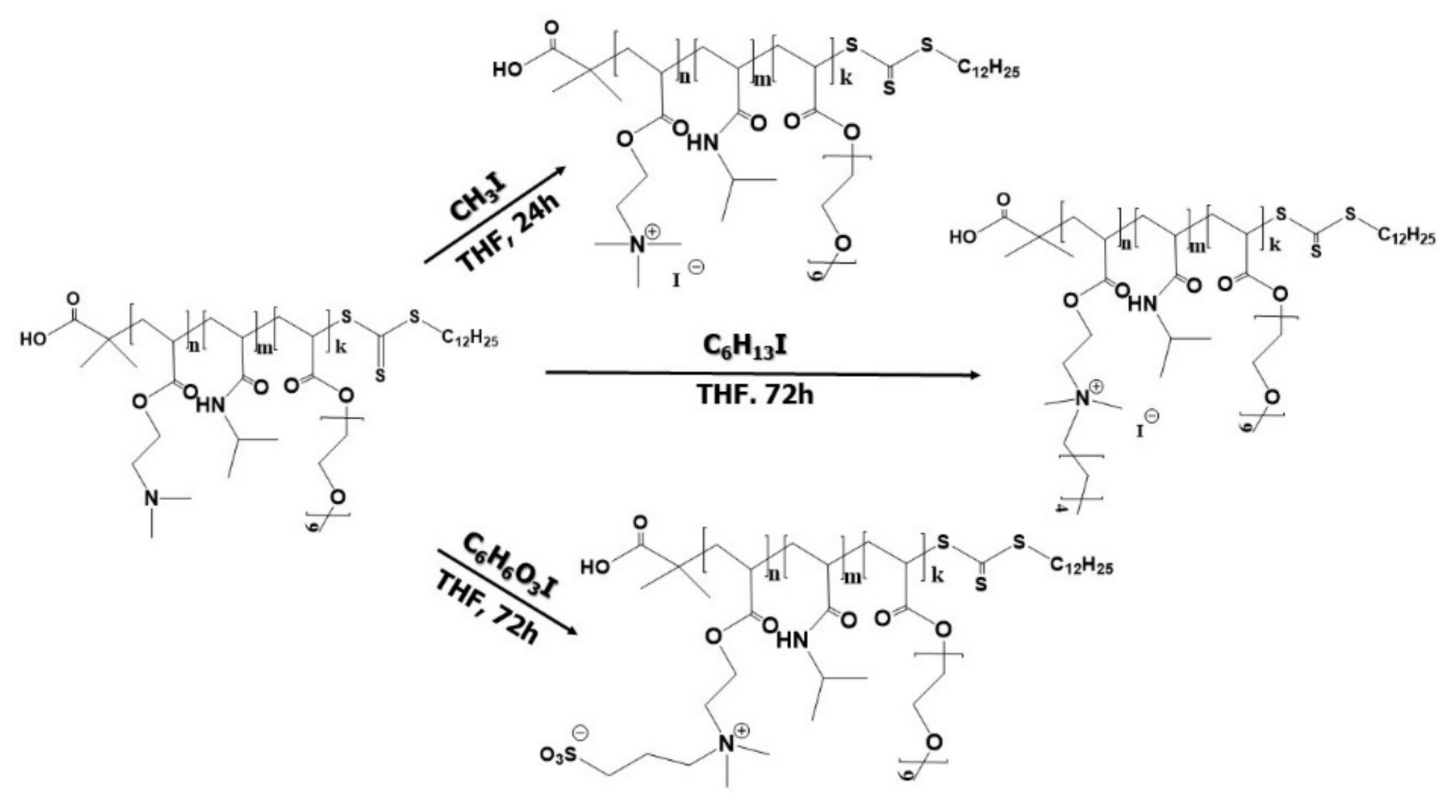
Chemical modification reactions of the amino-based PDMAEA-b-PNIPAM-b-POEGA triblock terpolymer.

Moreover, chemical modifications of the PDMAEA block were carried out via post-polymerization quaternization or sulfobetainization reactions so as to convert the tertiary amine groups of DMAEA units to their quaternary amine analogues and thus to produce strong cationic triblock polyelectrolytes or zwitterionic triblock polyampholyte, consisting of PNIPAM as the temperature-responsive block. It should be noted that the targeted composition of the amino-based triblock terpolymer and especially the higher amount of the POEGA block was designed in order to overcome solubilization barriers in aqueous media of chemically converted triblock terpolymers. Two alkyl halides with different alkyl chain length, i.e., iodomethane (CH_3_I) and 1-iodohexane (C_6_H_13_I), were used as the quaternizing agents in order to produce Q_1_PDMAEA_20_-b-PNIPAM_11_-b-POEGA_18_ and Q_6_PDMAEA_20_-b-PNIPAM_11_-b-POEGA_18_ quaternized triblock terpolymers of variable hydrophobicity. Additionally, 1,3 propanesultone (C_3_H_6_O_3_S) was utilized as the sulfobetainization agent to give SPDMAEA_20_-b-PNIPAM_11_-b-POEGA_18_ zwitterionic triblock terpolymers. The post-polymerization modifications taking place on the tertiary amine group of the PDMAEA_20_-b-PNIPAM_11_-b-POEGA_18_ triblock terpolymer are illustrated in Scheme 2. Proper stoichiometric calculations were performed such that the amino-based PDMAEA_20_-b-PNIPAM_11_-b-POEGA_18_ triblock terpolymers were modified at 100% utilizing quaternizing and zwitterionic agents. Three different chemical modification reactions of PDMAEA block were carried out in order to investigate the effect of quaternization agents on the thermoresponsive behavior of the terpolymers originating from the PNIPAM block and on the overall self-assembling properties of chemically modified triblock terpolymers in aqueous media. After the chemical modifications, the solubility of the PDMAEA block in water increases in the case of quaternization with iodomethane due to the presence of permanent cationic charges on the amine group, and thus the PDMAEA block converts to strong cationic polyelectrolyte. Its hydrophilicity decreases in the case of quaternization with iodohexane because of the existence of hydrophobic alkyl side chains on the Q_6_PDMAEA block [64]. Upon sulfobetainization reaction with 1,3-propanesultone, the solubility of the PDMAEA block in aqueous media decreases, as it is known from the literature that polyampholytes are less soluble in water [65]. Nevertheless, in all types of functionalization, the solubility of PDMAEA block is independent of solution pH.

**Figure 2:**
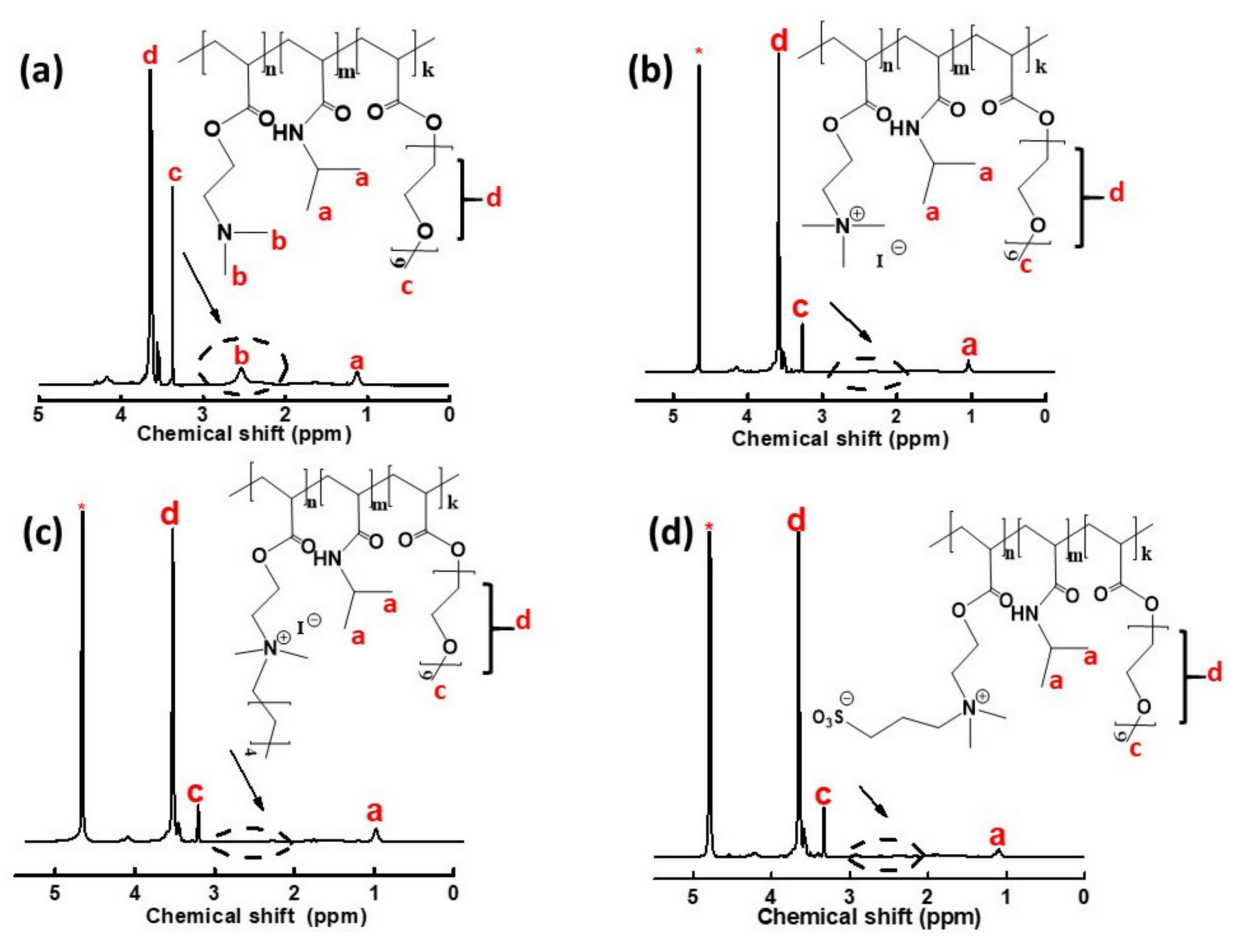
^1^H-NMR spectra for (**a**) PDMAEA_20_-b-PNIPAM_11_-b-POEGA_18_ (in CDCl_3_), (**b**) QPDMAEA_20_-b-PNIPAM_11_-b-POEGA_18_ (in D_2_O), (**c**) Q_6_PDMAEA_20_-b-PNIPAM_11_-b-POEGA_18_ (in D_2_O), and (**d**) SPDMAEA_20_-b-PNIPAM_11_-b-POEGA_18_ (in D_2_O).

The determination of composition for the precursor PDMAEA_20_-b-PNIPAM_11_-b-POEGA_18_ triblock terpolymer, as well as the verification of the chemical structure of all synthesized (amine and quaternized) triblock terpolymer analogues, were accomplished by ^1^H-NMR measurements. In Figure 2a, the ^1^H-NMR spectrum of the PDMAEA_20_-b-PNIPAM_11_-b-POEGA_18_ triblock terpolymer in CDCl_3_ is presented. It is worth noting that the composition of the amino-based triblock terpolymer calculated from the characteristic peaks of each block are close to stoichiometric calculations, after taking into account the yields of each synthesis step, as well as the quantity of each monomer utilized. In particular, the spectral peaks of terpolymer used for the calculations of composition are described in the following: the characteristic peak of PDMAEA block is at 2.26 ppm (b) [60,64], which corresponds to the –CH_3_ protons of the tertiary amine group and the corresponding peaks for the PNIPAM and POEGA blocks are at 1.2 ppm (a, methyl protons of N-isopropyl group of NIPAM) and at 3.63 ppm (d, methylene protons of oligo ethylene glycol side chains of OEGA) respectively [63,66].

Figure 2b–d shows the ^1^H-NMR spectra of the chemically modified triblock terpolymers. For all modified terpolymers, it can be observed that the peak b of the –CH_3_ proton attached to the tertiary amino group disappeared, evidencing the formation of quaternary ammonium group in all cases, as also reported in the literature [60,66,67]. Indeed, as observed in other studies [62,67], the characteristic peak of the methyl protons attached to the quaternary ammonium groups appears in the range of 3.1–3.5 ppm, depending on the quaternizing agent. However, in the above spectra, the characteristic peaks of the protons of the quaternary ammonium groups are not visible due to the presence of the POEGA block in the terpolymers (overlap with the corresponding peaks of methyl and methylene protons of the POEGA side chains appears in the range of 3.1–3.6 ppm).

It has been reported in the literature [62,65,67] that the quaternization reactions utilized here are quantitative, and the tertiary amine groups convert to the quaternary amine group at almost 100% yields, as confirmed after conducting ^1^H-NMR measurements. Consequently, the successful chemical modifications of the amine group on the side chain of PDMAEA block with quaternizing or sulfobetanizing agents were confirmed by the above-mentioned ^1^H-NMR experiments. The composition and the molecular weights of all quaternized triblock terpolymers were estimated utilizing the obtained molecular characteristics of the amino-based PDMAEA_20_-b-PNIPAM_11_-b-POEGA_18_ tribock terpolymer, as determined by SEC and ^1^H-NMR measurements. All calculations were made after taking into account that the reactions are almost quantitative. The obtained molecular characteristics for all synthesized triblock terpolymers are listed in Table 1.

### 3.2. Aqueous Solution Properties of Triblock Terpolymers 

Studies on the self-assembly behavior of the novel triblock terpolymers (amino and chemically modified analogs) in the aqueous medium were conducted utilizing light scattering techniques and fluorescence spectroscopy. Taking into account the sequence and the nature of the blocks, it is expected that the amino-based triblock terpolymer should form nanoassembled structures in aqueous solutions responding to temperature changes due to variations in the solvation/conformational state of the thermoresponsive PNIPAM block and to solution pH alterations because of variations in the hydration/conformational state of the pH-responsive PDMAEA block (pKa = 8.41), due to protonation at neutral and low pH and deprotonation at basic pH. The block sequence indicates that as the temperature increases above the nominal LCST of the PNIPAM block, which gradually obtains a less hydrophilic character [47] (not utterly hydrophobic due to interactions with water molecules even at higher temperatures), the triblock terpolymer should self-assemble into nanostructures where the PNIPAM middle block should occupy the core domains, and the PDMAEA as well as POEGA blocks should constitute the mixed hydrophilic shell of the aggregates formed. It is anticipated also that the chemically modified triblock terpolymers should form nanostructures in aqueous media where the PNIPAM middle blocks should constitute the inner part and the chemically modified PDMAEA and POEGA blocks should constitute the outer part as solution temperature increases above the nominal LCST of PNIPAM. Some deviations from this general picture are expected in the case of the Q_6_PDMAEA_20_-b-PNIPAM_11_-b-POEGA_18_ terpolymer, since in this case, the C6 side chains connected to a positively charged nitrogen confer hydrophobicity in the otherwise hydrophilic cell of the nanostructures. The triblock terpolymers were readily soluble in aqueous media at room temperature.

Light-scattering techniques are a powerful tool in order to get information about the size and morphology of the formed aggregates. Dynamic (DLS) and static (SLS) light scattering measurements for all synthesized triblock terpolymers were conducted so as to determine the weight average apparent molecular weights (M_w_, _app_), the aggregation number (N_agg_), and the apparent hydrodynamic radius (R_h_) of the formed polymeric nanoassemblies in aqueous solutions as a function of temperature. Moreover, the morphology of the self-assembled nanostructures can be determined using the R_g_/R_h_ ratio extracted by the combination of static and dynamic light scattering. The obtained results from light scattering measurements at 25 °C and 45 °C (above the LCST) are listed in Table 2.

As it can be observed in Table 2, the obtained M_w, app_ values are much higher than the molecular weight of single chains (M_w_) determined by SEC for the PDMAEA_20_-b-PNIPAM_11_-b-POEGA_18_ amino-based terpolymer and the theoretical M_w_ calculated assuming 100% conversion for chemically modified terpolymers, indicating the presence of aggregation phenomena even at ambient temperature. Bearing in mind that the overall molecular weights of the triblock terpolymers are relatively low, the formation of aggregates even at room temperatures may be attributed to the intramolecular hydrogen bonding between the NIPAM units and the comonomers, and interactions between the carboxylate terminal groups contained in CTA and the segments of triblock terpolymers. This latter behavior is also supported by the aggregation numbers (N_agg_) calculated as the M_w, app_/M_w_ ratio, which are in the range of 9–47 at 25 °C (temperature below the nominal LCST of the PNIPAM block). It is worth noting that similar observations are also mentioned in other studies [48,63] for PNIPAM-based block copolymers’ self-assembling behavior in water at room temperatures where the hydrogen bond interactions between the monomeric NIPAM units as well as the influence of terminal end group at the polymer chains are reported as the possible reason for this behavior. At 45 °C (temperature above the nominal LCST of the PNIPAM block), the N_agg_ values range from 115 to 429, showing that the overall aggregation tendency is considerably higher on account of the thermoresponsiveness of the PNIPAM block that gets a less hydrophilic character above the LCST value, which is due to intramolecular hydrophobic interactions between the isopropyl groups attached to the NIPAM units. However, it could be noticed that the aggregation numbers at both temperatures (25 °C and 45 °C) for Q_1_PDMAEA_20_-b-PNIPAM_11_-b-POEGA_18_ are significantly lower that the amine-based triblock terpolymer, and this fact should be associated with the permanent positive charges on the triblock terpolymer chains contributing to the increased solubility and therefore to the decreased aggregation tendency in aqueous media. On the contrary, the Q_6_PDMAEA_20_-b-PNIPAM_11_-b-POEGA_18_ triblock terpolymer displays a rather different aggregation behavior at both temperatures (comparatively higher M_w, app_ and N_agg_ values at both 25 °C and 45 °C, Table 2) compared to the Q_1_PDMAEA_20_-b-PNIPAM_11_-b-POEGA_18_, which is most probably due to the existence of hydrophobic alkyl (C_6_H_13_) side chains attached to the DMAEA units, leading to secondary aggregation phenomena via hydrophobic interactions between the coronas of the formed aggregates even at room temperature, as also reported in the literature [62]. In the case of SPDMAEA_20_-b-PNIPAM_11_-b-POEGA_18_, the aggregation tendency also may be enhanced by the formation of intramolecular and intermolecular electrostatic interactions between the zwitterionic sulfobetaine groups attached to the DMAEA segments [68].

#### 3.2.1. Effects of Temperature and pH on the Self-Assembly Behavior of PDMAEA-b-PNIPAM-b-POEGA Terpolymers

Taking into consideration the nature of the blocks in the triblock terpolymer and in particular the combination of PDMAEA (pH-responsive block), PNIPAM (thermoresponsive block), and POEGA (hydrophilic block) in the same macromolecule, the investigation of the aqueous solution properties of such a terpolymer is quite appealing. The effects of temperature and pH on the self-assembly process in water of the triblock terpolymer were examined by varying one of these stimuli simultaneously or individually. For this purpose, light scattering (DLS and SLS) measurements were performed at three different solution pHs (acidic, neutral, and basic) and conducting measurements by increasing solution temperature from 25 to 55 °C, by steps of 5 °C.

**Figure 3:**
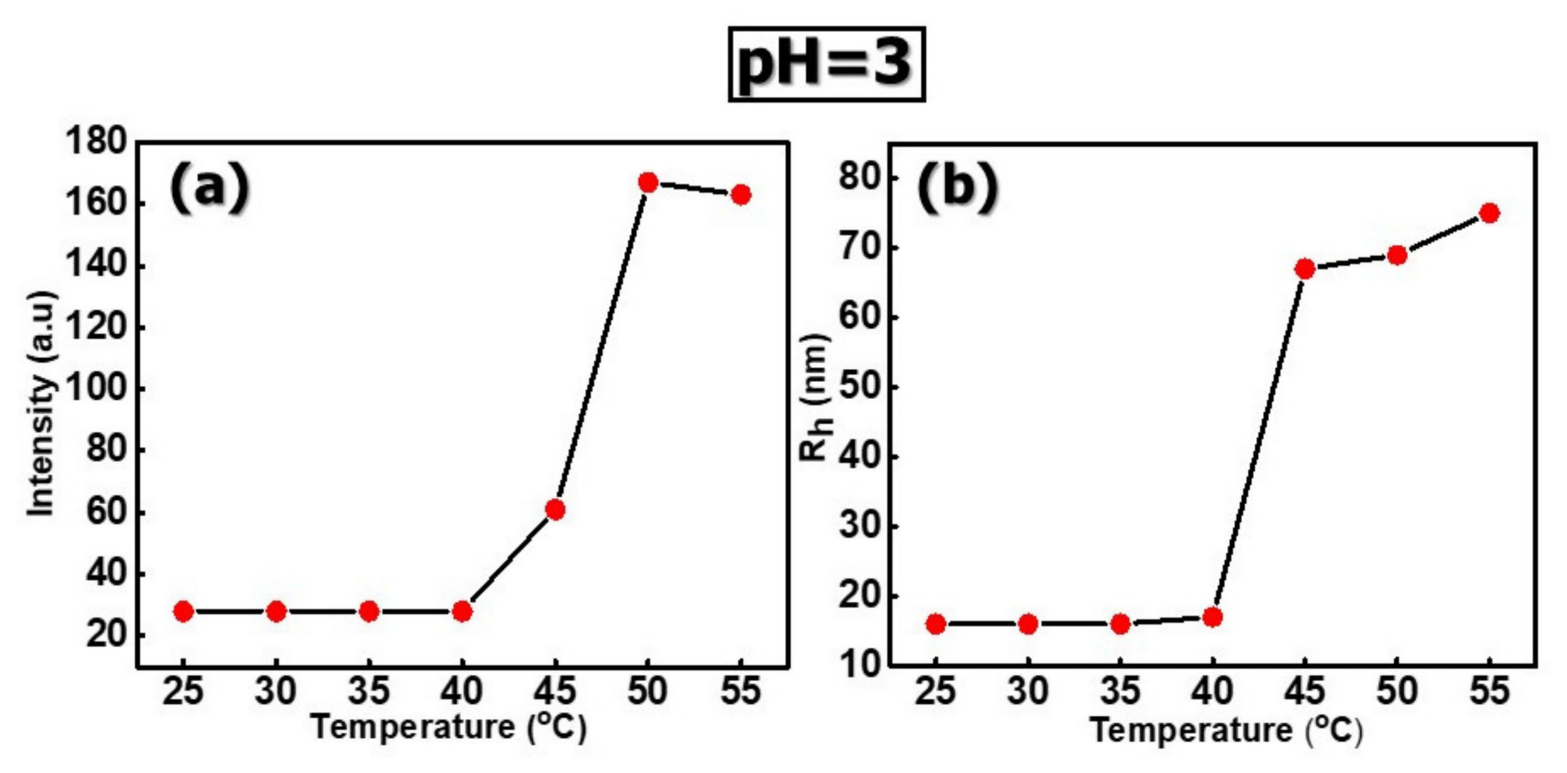

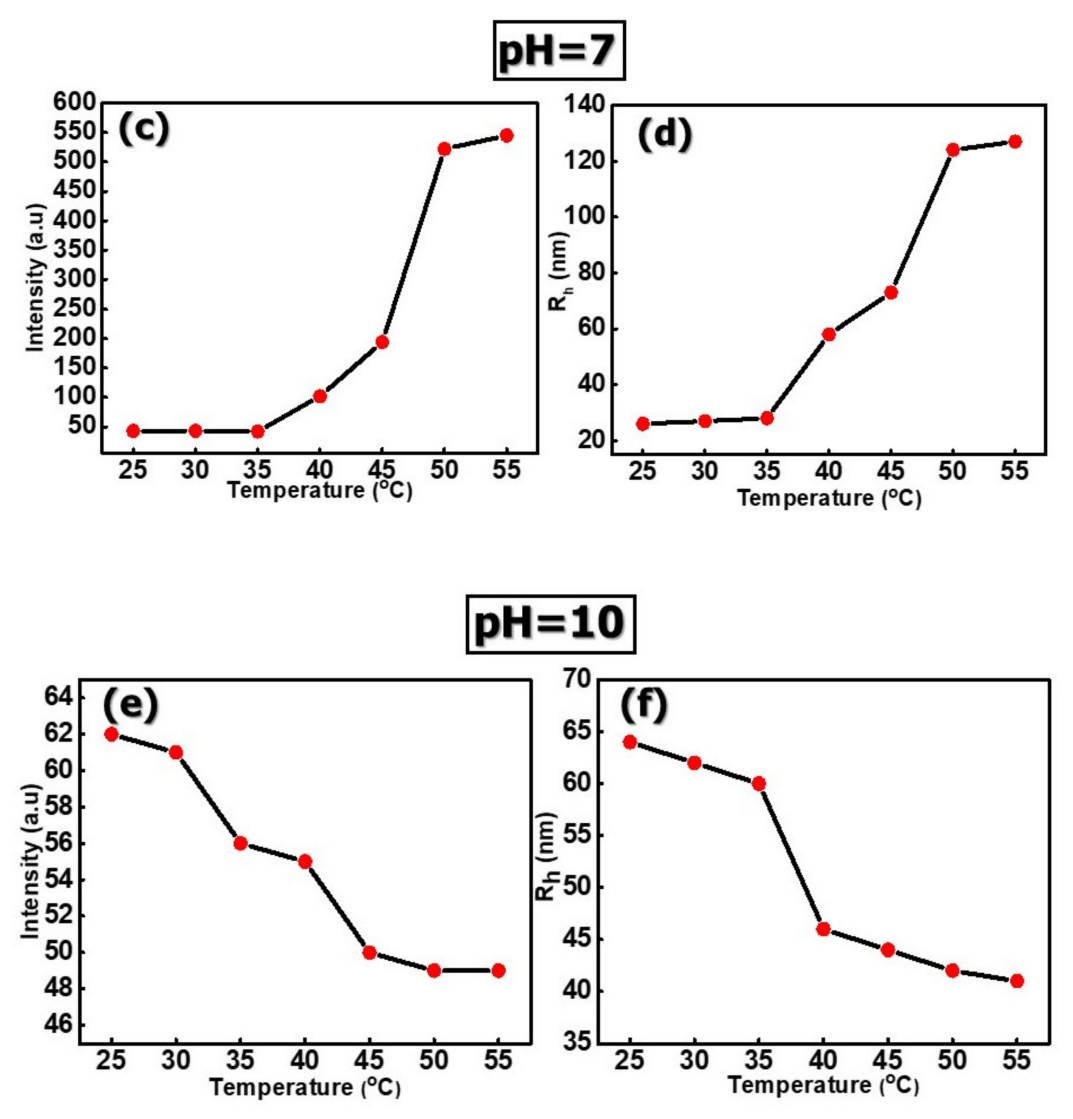
The dependence of scattering intensity and R_h_ on temperature at pH = 3 (**a**,**b**), pH = 7 (**c**,**d**), and pH = 10 (**e**,**f**) for the PDMAEA_20_-b-PNIPAM_11_-POEGA_18_ triblock terpolymer in aqueous media (at an angle of 90°).

Figure 3 depicts DLS results for the dependence of scattered intensity and R_h_ on temperature at acidic (a,b), neutral (c,d), and basic (e,f) pH solutions. It can be observed that the self-assembly behavior of the PDMAEA_20_-b-PNIPAM_11_-POEGA_18_ triblock terpolymer is affected by variations in pH and temperature. For both acidic and neutral solutions, the scattering intensity and the hydrodynamic radius (R_h_) values increase as the temperature solution increases, indicating an increase in both mass and size of the formed aggregates. However, it can be noticed that in acidic solution, the LCST is moved to higher temperatures. The latter can be explained by the fact that at pH = 3, the PDMAEA chains are considerably hydrophilic, because their tertiary amine groups are fully protonated and therefore the overall hydrophilicity of the system increases, displacing the LCST phase transition at higher temperatures. At neutral pH, the PDMAEA block is quite hydrophilic because it is partially protonated, and therefore, the thermoresponsiveness of the PNIPAM block has a stronger effect on the self-assembly process. Furthermore, it is worth noting that the almost linear increase in the hydrodynamic radius of the formed aggregates is a consequence of the thermoresponsive nature of low-molecular weight PNIPAM block, as also reported in other studies [63,69]. In contrast, at basic pH, the terpolymer displays a rather different self-assembly behavior where both the scattered intensity and R_h_ values decrease as the solution temperature increases. At basic pH, the deprotonation of the PDMAEA block renders it less hydrophilic; this feature dictates the self-organization in the system, and the formation of larger aggregates is taking place (judging from the intensity and hydrodynamic radius values). The decrease in hydrodynamic radius of the formed aggregates by increasing temperature can be correlated with the shrinking of PNIPAM chains enhanced by the hydrophobic collapse of PDMAEA chains, which apparently leads to a partial dissociation of the initial aggregates. It can be concluded that at basic pH due to the deprotonation of the PDMAEA block, the thermoresponsiveness of the PNIPAM block affects the self-assembly procedure in a more acute way. 

**Figure 4:**
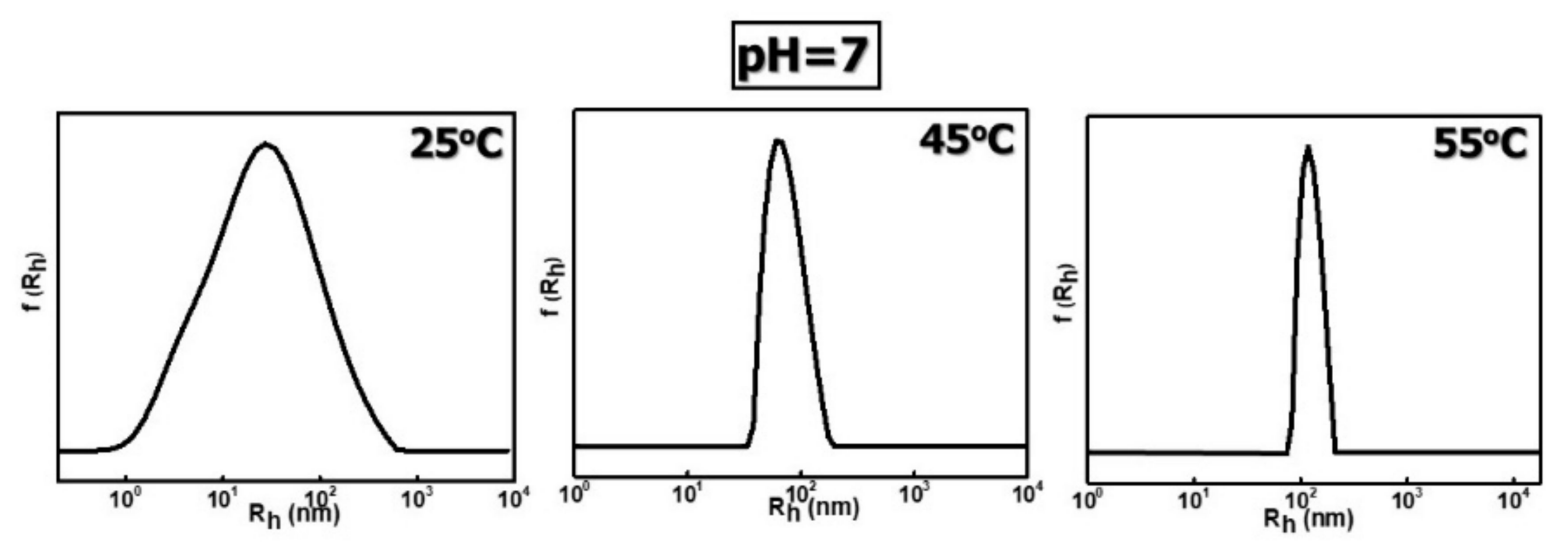
Hydrodynamic radius distributions from Contin analysis for the PDMAEA_20_-b-PNIPAM_11_-b-POEGA_18_ triblock terpolymer in aqueous solutions at neutral pH and at selected temperatures (at an angle of 90°).

The hydrodynamic radius distributions from Contin analysis extracted by DLS measurements for the PDMAEA-b-PNIPAM-b-POEGA triblock terpolymer at pH = 7 and at three different temperatures are presented in Figure 4.

As observed in Figure 4, the obtained DLS results revealed the formation of monodisperse aggregates by elevation of the temperature above the LCST of the PNIPAM block. At 25 °C, there is a broad peak with an R_h_ value around 26 nm, indicating the formation of aggregates even at ambient temperature, due to the overall low-molecular weight of terpolymer, the hydrogen bonding interactions between the NIPAM units, as well as hydrogen bonding interactions between the terminal carboxylate groups contained in the chain transfer agent, with the segments of the blocks. The aforementioned behavior at room temperature is consistent with previous findings for low-molecular weight PNIPAM-based copolymers [63]. Increasing the solution temperature, narrow and symmetrical peaks can be observed, pointing out the participation of all polymeric chains in the formation of one type of nanostructure in the solution due to the thermally induced aggregation phenomena driven by the thermoresponsive character of the PNIPAM block. Above the phase-transition temperature, the PNIPAM block experiences conformational variations and changes in the solvation state. As a result, it becomes less hydrophilic (lessened hydrogen bonding interactions with water molecules), leading to the formation of more compact aggregates. Moreover, it should be noted that at 25 °C, the obtained R_h_ value is rather similar compared to the calculated length (L = 12) of the completely extended macromolecular chain (L = the number of the monomeric units of each block multiplied by 0.254, which corresponds to nearly the half micellar diameter for spherical core–shell micelles) [65], indicating that the formed aggregates may have micellar-like core–shell structures. Above the phase transition temperature, the formation of vesicular-like structures is a possible scenario since the hydrodynamic radius (at 45 °C the R_h_ is 73 nm and at 50 °C the R_h_ is 127 nm) is much higher than the block terpolymer counter length, and also, the very low size polydispersity indices (PDI < 0.1) obtained for the aggregates indicates further the formation of vesicular-like morphologies. 

Additionally, at both pH = 3 (Figure S1) and pH = 10 (Figure S2), the hydrodynamic size distributions become symmetric and narrower as the temperature increases. It is worth mentioning that the obtained peaks for the acidic pH are broader compared to the hydrodynamic size distributions at basic pH, which is most probably due to the negatively charged PDMAEA block that becomes less hydrophilic, leading to the formation of more compact aggregates. 

**Figure 5:**
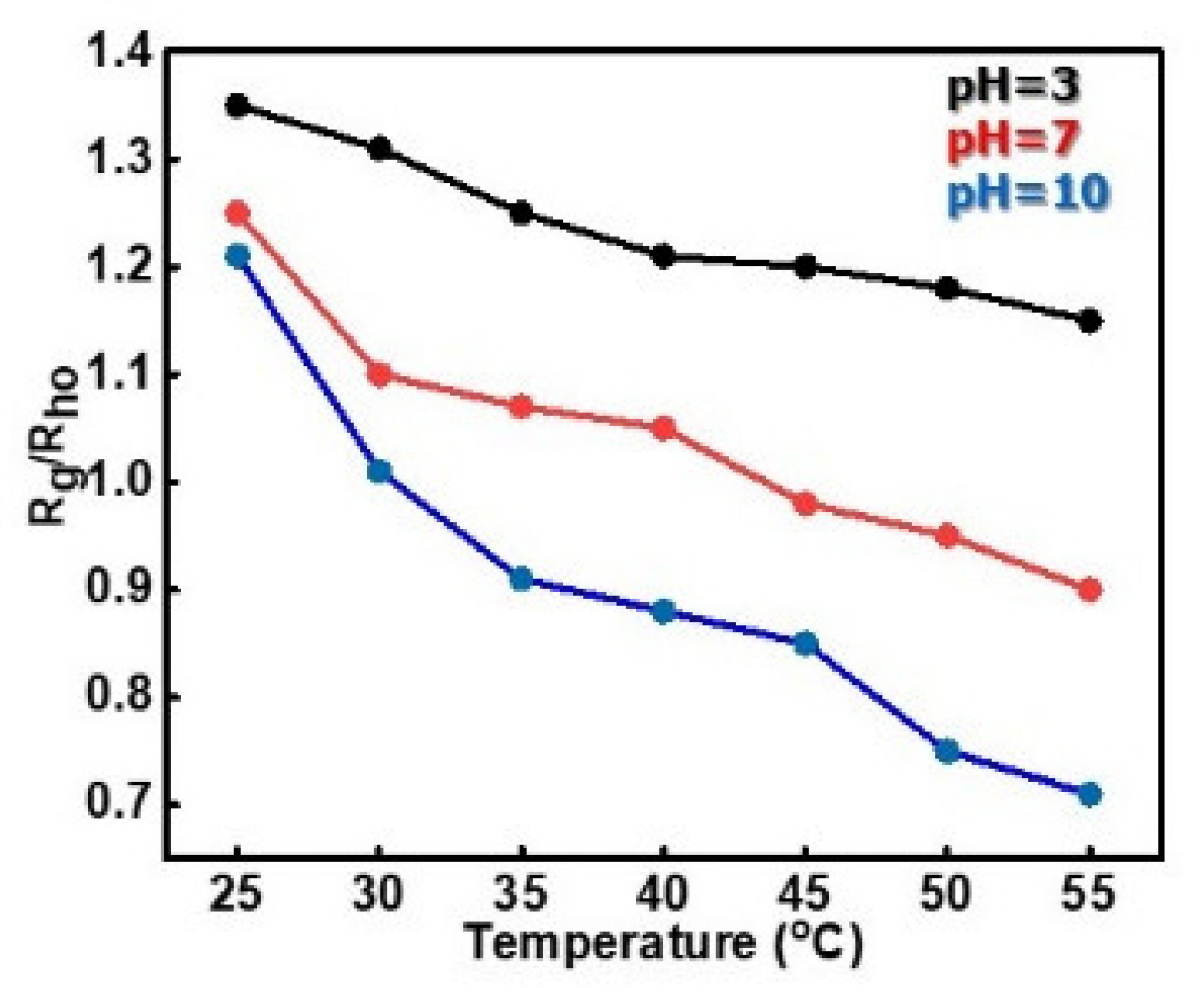
R_g_/R_ho_ ratio values at acidic, neutral, and basic pH solution as a function of temperature for the PDMAEA_20_-b-PNIPAM_11_-b-POEGA_18_ triblock terpolymer.

From SLS/DLS measurements, the R_g_/R_ho_ ratio values extracted are in the range of 0.7–1.35 for the triblock terpolymer aqueous solutions at three different pH solutions as a function of temperature (Figure 5).

The R_g_/R_ho_ ratio values can give significant details about the morphology/structure of the formed nanostructures. As it is reported in the literature, the corresponding R_g_/R_ho_ ratio values for spherical micellar structures (core–shell morphology) are in the range of 0.7–0.9, and for vesicular structures, they are in the range of 0.9–1.9 [70]. Therefore, the formed nanosized particles are expected to have spherical structures in the cases that the determined R_g_/R_ho_ ratio values are close to 0.8 and maybe vesicular structures in the cases that the determined R_g_/R_ho_ ratio values are above 0.9. However, it should be mentioned that unambiguous conclusions about the morphology of the formed aggregates cannot be made from SLS measurements alone. For acidic pH, the R_g_/R_ho_ ratio values are in the range of 1.15–1.35, and there are no important variations with increasing the temperature, which is presumably due to the high hydrophilicity of the fully protonated PDMAEA block. For neutral and basic pH, there are significant changes in the R_g_/R_ho_ ratio values, which are in the range of 0.7–1.25. This may be correlated with the fact that at neutral pH solution, the PDMAEA blocks are partially protonated, and at basic pH solution, they are fully deprotonated, which is a feature that seems to have an intense effect on the structure/morphology of the aggregates that are self-assembled in the corresponding aqueous solutions by increasing temperature. However, other morphologies cannot be evaluated by SLS data, and the above-mentioned results are only an indication about the morphology of the nanostructures due to the absence of confirming evidence from imaging techniques.

From the aforementioned results, it can be deduced that the PDMAEA_20_-PNIPAM_11_-POEGA_18_ triblock terpolymer forms well-defined aggregates in response to pH changes due to the pH-responsive PDMAEA block and temperature variations, even though the length of the thermoresponsive PNIPAM block is rather low. 

#### 3.2.2. Self-Assembly Properties of Chemically Modified PDMAEA-b-PNIPAM-b-POEGA Triblock Terpolymers in Aqueous Media as a Function of Temperature

The self-assembly behavior of chemically modified triblock terpolymers was also investigated using light scattering techniques. In Figure 5, the dependence of scattering intensity and hydrodynamic radius on temperature for chemically modified triblock terpolymers in aqueous media is depicted.

**Figure 6:**
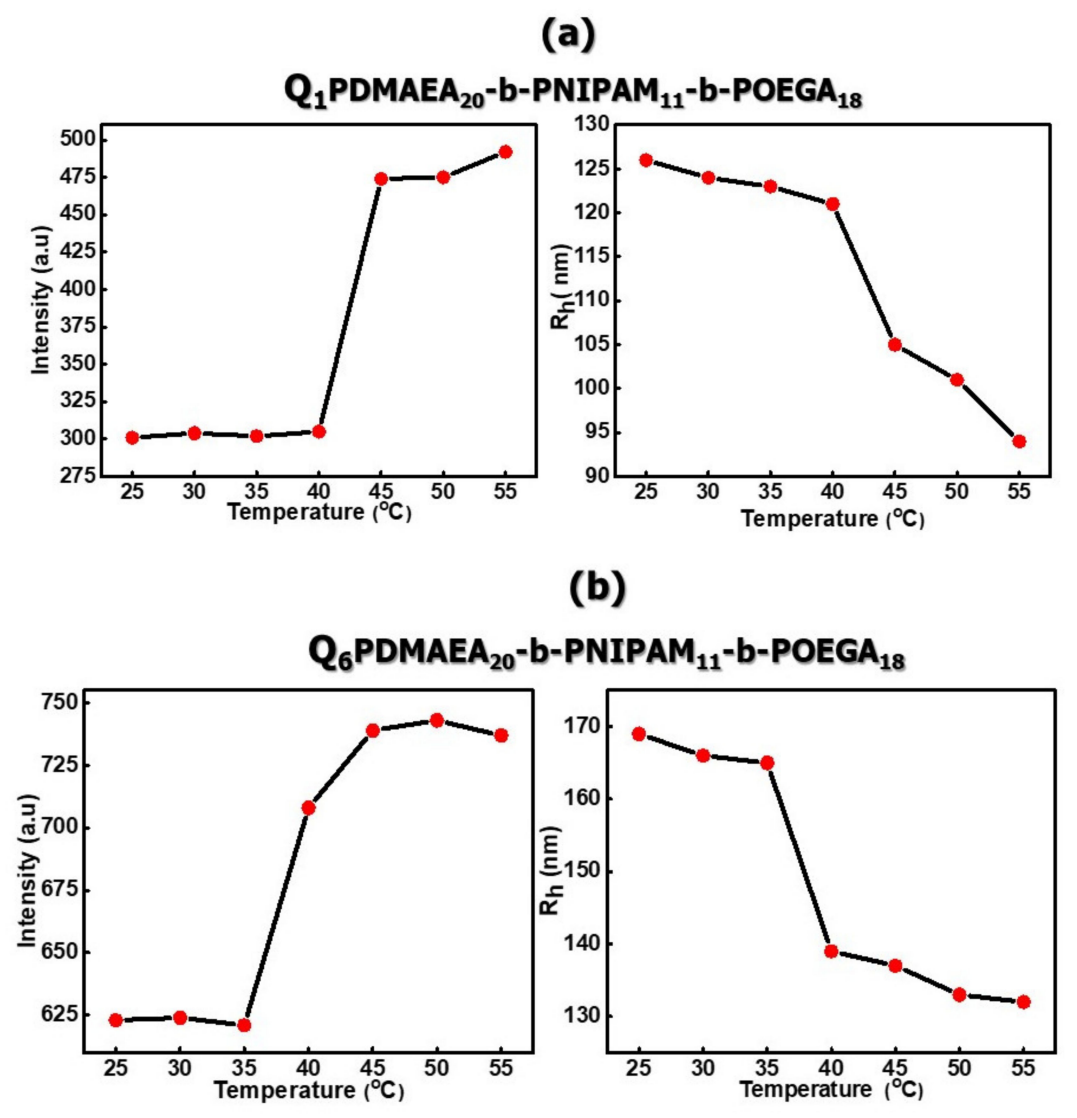

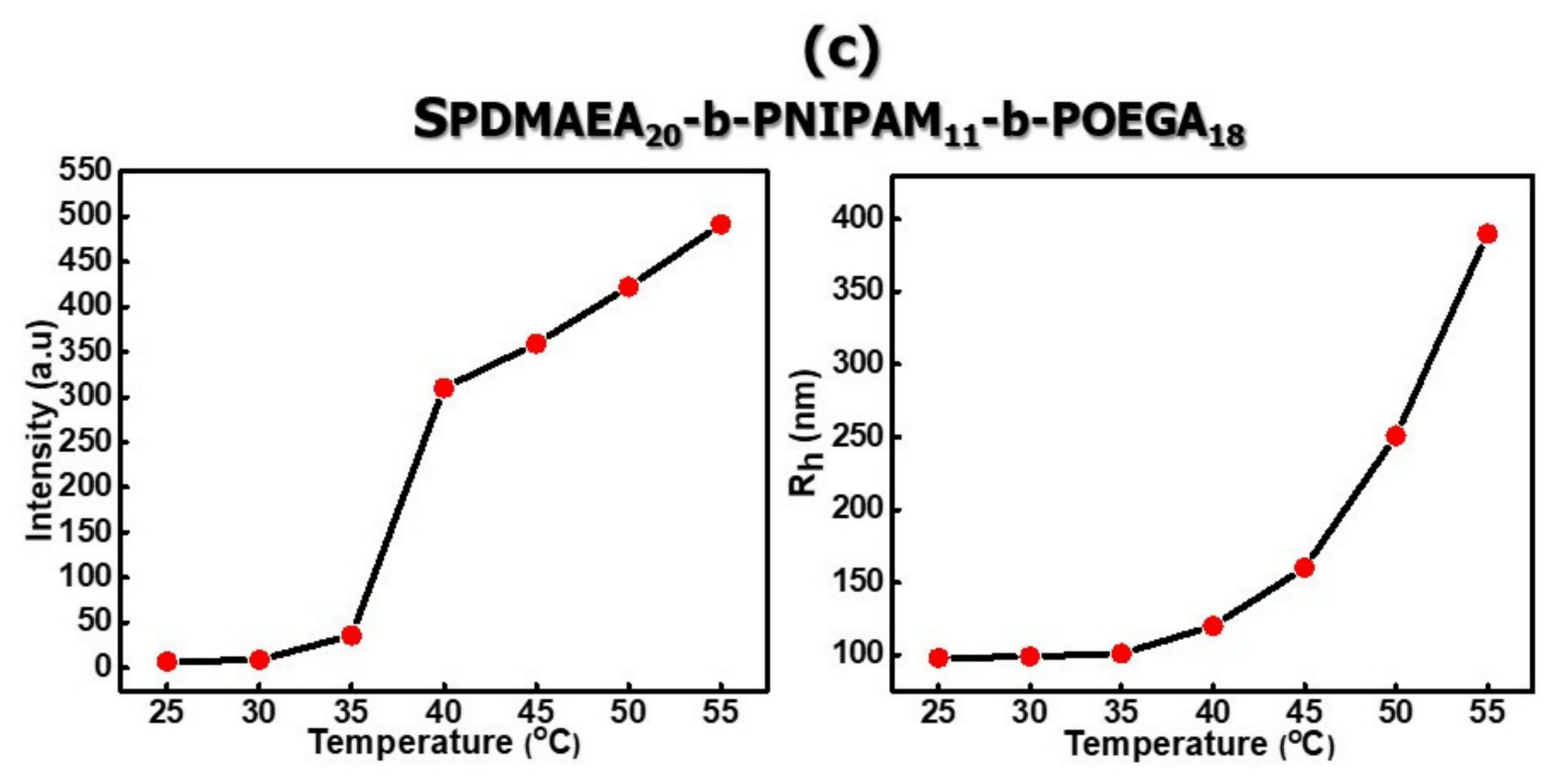
The dependence of scattered intensity and hydrodynamic radius on temperature for Q_1_PDMAEA_20_-b-PNIPAM_11_-b-POEGA_18_ (**a**), Q_6_PDMAEA-b-PNIPAM_11_-b-POEGA_18_ (**b**), and SPDMAEA_20_-b-PNIPAM_11_-b-POEGA_18_ (**c**) in aqueous solutions at pH = 7 and c = 1 × 10^−3^g/mL. (measurements obtained at a 90° angle). The hydrodynamic radius of SPDMAEA_20_-b-PNIPAM_11_-b-POEGA_18_ is the average values extracted from the cumulants method.

For the Q_1_PDMAEA_20_-b-PNIPAM_11_-b-POEGA_18_ (Figure 6a) and Q_6_PDMAEA_20_-b-PNIPAM_11_-b-POEGA_18_ (Figure 6b) cationic triblock terpolymers, the scattering intensity values increase and R_h_ values decrease as the solution temperature increases above the LCST-like phase-transition temperature. However, it can be observed that the LSCT-like transition of the Q_1_PDMAEA_20_-b-PNIPAM_11_-b-POEGA_18_ cationic terpolymer is at a higher temperature compared to the LCST-like transition of the Q_6_PDMAEA_20_-b-PNIPAM_11_-b-POEGA_18_ cationic terpolymer. The existence of long hydrophobic side chains (C_6_H_13_) attached to the PDMAEA block of the Q_6_PDMAEA_20_-b-PNIPAM_11_-b-POEGA_18_ terpolymer decrease the overall hydrophilicity of the system, which explains the shift of the LSCT at lower temperatures. Moreover, it can be noticed that the Q_6_PDMAEA_20_-b-PNIPAM_11_-b-POEGA_18_ aggregates exhibit an R_h_ plateau above 40 °C, indicating a rather small diminution in the size compared to the hydrodynamic radius of Q_1_PDMAEA_20_-b-PNIPAM_11_-b-POEGA_18_ aggregates, which continuously decrease in the whole temperature range. This may be explained by the fact that in the case of the Q_6_PDMAEA_20_-b-PNIPAM_11_-b-POEGA_18_ terpolymer, secondary aggregation phenomena may take place due to the amphiphilic character of the formed aggregates shell. On the other hand, the SPDMAEA_20_-b-PNIPAM_11_-b-POEGA_18_ zwitterionic terpolymer (Figure 6c) displays a different self-assembly behavior as a function of temperature compared to its cationic counterparts. It should be mentioned that the hydrodynamic radius values in the whole temperature range are the average values extracted from the cumulants method. By increasing temperature, both scattered intensity and hydrodynamic radius increase, showing that the initially formed nanoassemblies increase in mass and size. Increasing the temperature, this behavior may be attributed not only to the shrinking of thermoresponsive PNIPAM blocks but also to the intramolecular and intermolecular electrostatic interactions between the zwitterionic groups attached to the PDMAEA blocks, leading to the formation of larger aggregates. In addition, it should be noted that the obtained hydrodynamic radius values for chemically modified triblock terpolymers are much higher compared to the amine-based triblock terpolymer. It is probable that the existence of positive or zwitterionic charges on the PDMAEA blocks results in the formation of larger nanoaggregates due to electrostatic repulsions of the modified amine groups and thus the extension of the PDMAEA blocks. This is especially true in the case of the zwitterionic terpolymer due to the low solubility of polyzwitterions in aqueous media of low ionic strength.

**Figure 7:**
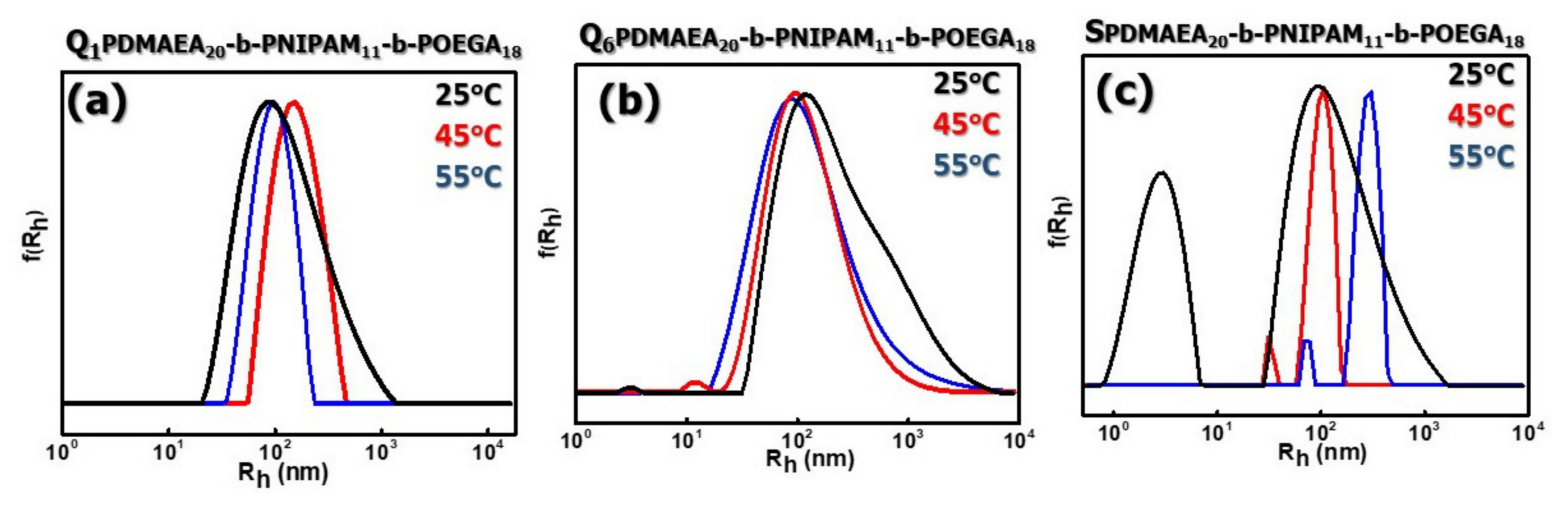
Hydrodynamic radius distributions for Q_1_PDMAEA_20_-b-PNIPAM_11_-b-POEGA_18_ (**a**), Q_6_PDMAEA_20_-b-PNIPAM_11_-b-POEGA_18_ (**b**), and SPDMAEA_20_-b-PNIPAM_11_-b-POEGA_18_ (**c**) modified triblock terpolymers at 25 °C, 45 °C, and 55 °C. (angle of 90°).

The hydrodynamic radius distributions for the chemically modified triblock terpolymers at selected temperatures are presented in Figure 7.

By increasing the temperature, the Q_1_PDMAEA_20_-b-PNIPAM_11_-b-POEGA_18_ cationic triblock terpolymer seems to self-assemble into well-defined aggregates (narrow and symmetric size distributions) in aqueous media, mainly due to conformational variations in the thermoresponsive PNIPAM block. On the contrary, the size distributions for the Q_6_PDMAEA_20_-b-PNIPAM_11_-b-POEGA_18_ quaternized triblock terpolymer are broader, and the mean size of the formed aggregates are relatively larger, when the solution temperature rises above the LCST-like value, which is most probably because of the existence of hydrophobic alkyl side chains in the PDMAEA block. This is the dominant chemical difference compared to the Q_1_PDMAEA_20_-b-PNIPAM_11_-b-POEGA_18_ cationic triblock terpolymer that dictates the observed behavior. Hydrophobic interactions between the shells of the formed aggregates lead to secondary aggregation phenomena and result in the formation of less well-defined nanostructures in terms of hydrodynamic radius distributions, as a result of conformational restrictions imposed on the terpolymer chains within the aggregates. In the case of the SPDMAEA_20_-b-PNIPAM_11_-b-POEGA_18_ zwitterionic triblock terpolymer, at 25 °C, two peaks can be obviously observed, showing the presence of two distinct aggregate structures in the aqueous solution. The first peak with R_h_ = 3 nm presumably refers to non-interacting isolated terpolymer chains and the second one with R_h_ = 98 nm refers to supramolecular aggregates, which can be formed due to hydrogen bonding interactions between the NIPAM units, as well as electrostatic interactions between the zwitterionic groups attached to the PDMAEA blocks. By increasing temperature, the first peak becomes small in the terms of intensity, the second peak becomes extremely narrow and symmetrical, indicating the participation of nearly all polymer chains in the formation of well-defined nanostructures, which is driven by the collapse of PNIPAM chains and enhanced by the intermolecular and intramolecular electrostatic interactions between the zwitterionic groups in the PDMAEA blocks.

Moreover, it is worth mentioning that the R_g_/R_ho_ ratio values extracted by SLS/DLS measurements are in the range of 0.98–1.45 for all the chemically modified triblocks and the temperature ranges studied, which indicate that the formed aggregates probably have spherical or vesicular-like morphologies based on the results extracted from SLS measurements. 

**Scheme 3:**
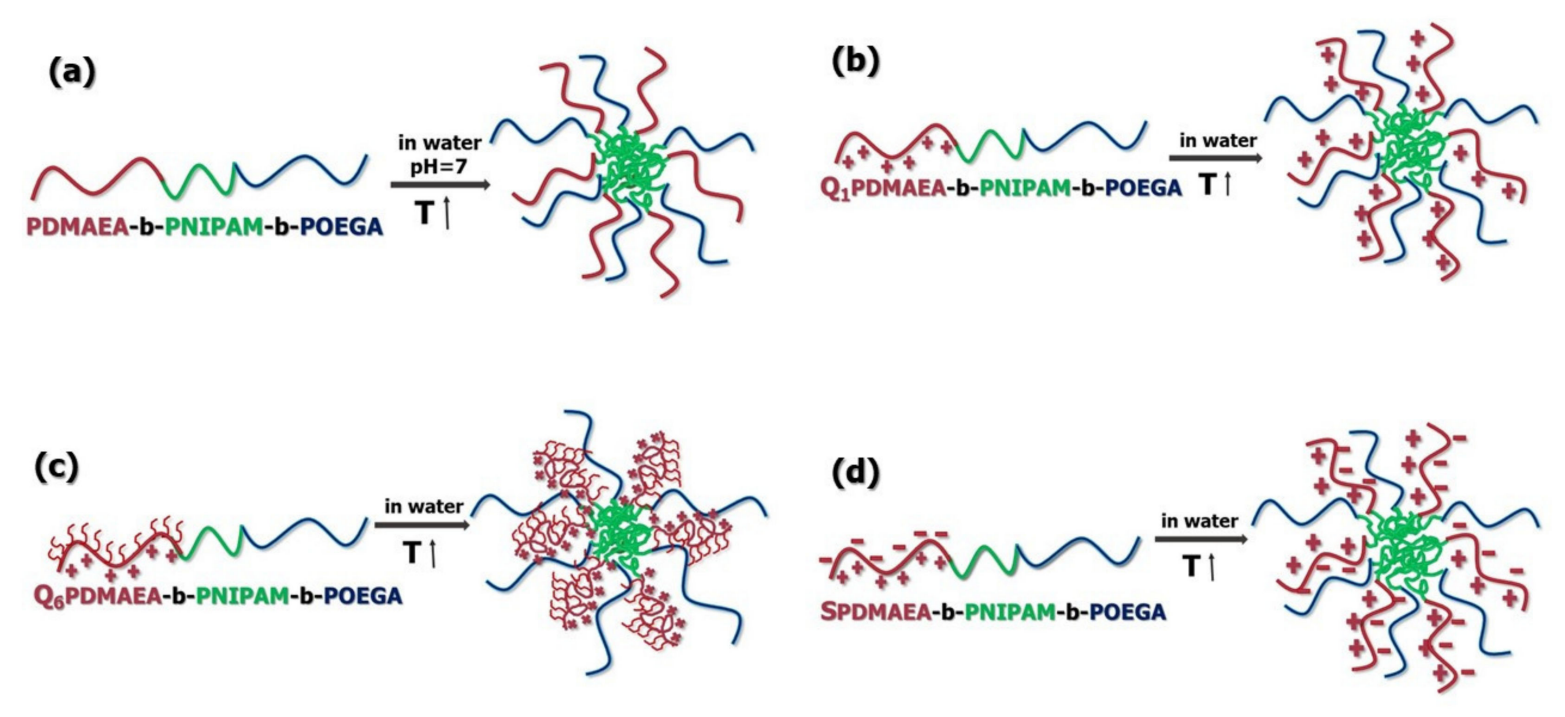
Graphical illustration of PDMAEA-b-PNIPAM-b-POEGA (**a**), Q_1_PDMAEA-b-PNIPAM-b-POEGA (**b**), Q_6_PDMAEA-b-PNIPAM-b-POEGA (**c**), and SPDMAEA-b-PNIPAM-b-POEGA (**d**) triblock terpolymers self-assembly behavior in water upon increasing the solution temperature.

A schematic illustration of triblock terpolymers (amino and chemically modified) self-assembly behavior in aqueous media is depicted in Scheme 3.

It can be concluded that as the temperature rises, the triblock terpolymers formed aggregates with probably spherical morphology where the PNIPAM block constitute the core domain and the PDMAEA/POEGA blocks form the mixed hydrophilic shell. In the case of the PDMAEA-b-PNIPAM-b-POEGA triblock terpolymer, the variations in solution pH affect the self-assembly procedure due to the presence of the pH-responsive PDMAEA block, which is protonated at low pH values and deprotonated at high pH values. At elevated temperatures, the Q_1_PDMAEA-b-PNIPAM-b-POEGA quaternized terpolymer and SPDMAEA-b-PNIPAM-b-POEGA sulfobetainized terpolymer self-assemble into well-defined nanostructured aggregates. In contrast, the hydrophobically modified Q_6_PDMAEA-b-PNIPAM-b-POEGA terpolymer displays less well-defined aggregates, which is presumably due to hydrophobic interactions in the shell that are derived by the hydrophobic alkyl side chains on the tertiary amine groups of the PDMAEA block. 

#### 3.2.3. Surface Charge and Micropolarity of the Formed Aggregates

The surface charge of the formed aggregates from amine-based and chemically modified triblock terpolymers was investigated by utilizing electrophoretic light scattering measurements, and the obtained results are listed in Table 2. As it can be noticed, at room temperature, the ζ_pot_ values for all samples are positive, since the PDMAEA blocks are partially protonated in the amine-based triblock terpolymer and fully protonated in the chemically converted triblock terpolymers. Upon the increasing temperature, the surface charges of the amine and quaternized triblock terpolymers aggregates increase. This increase should be attributed to the increased number of positive charges on the surface of the formed aggregates, which is enhanced by the increased aggregation number. However, in the case of the zwitterionic triblock terpolymer, at 45 °C, the ζ_pot_ value slightly decreases as the solution temperature rises, which is presumably due to the existence of SO_3_^−^ groups in the SPDMAEA blocks located in the shell of the aggregates. In addition, it can be observed that at 45 °C, the ζ_pot_ value of the Q_6_PDMAEA_20_-b-PNIPAM_11_-POEGA_18_ quaternized terpolymer presents a less positive surface charge than the Q_1_PDMAEA_20_-b-PNIPAM_11_-POEGA_18_ quaternized triblock terpolymer, which is most probably because of the presence of hydrophophic alkyl side chains (C_6_H_13_) on the Q_6_PDMAEA blocks, which may partially shield the positively charged amine groups.

Fluorescence spectroscopy (FS) experiments were performed in order to study the internal micropolarity of the formed polymeric aggregates in aqueous solutions using pyrene as the fluorescence probe. Pyrene is commonly utilized to investigate the internal micropolarity, since the I_1_/I_3_ ratio (the ratio of the first to the third vibronic peaks in the pyrene emission spectrum) is sensitive to the micropolarity variations, and particularly in less polar media, the I_1_/I_3_ ratio values decrease. In our study, the evaluation of the aggregate internal micropolarity was achieved by determining the I_1_/I_3_ ratio values at 25 °C and 45 °C, i.e., for comparison below and above LCST-like.

**Figure 8:**
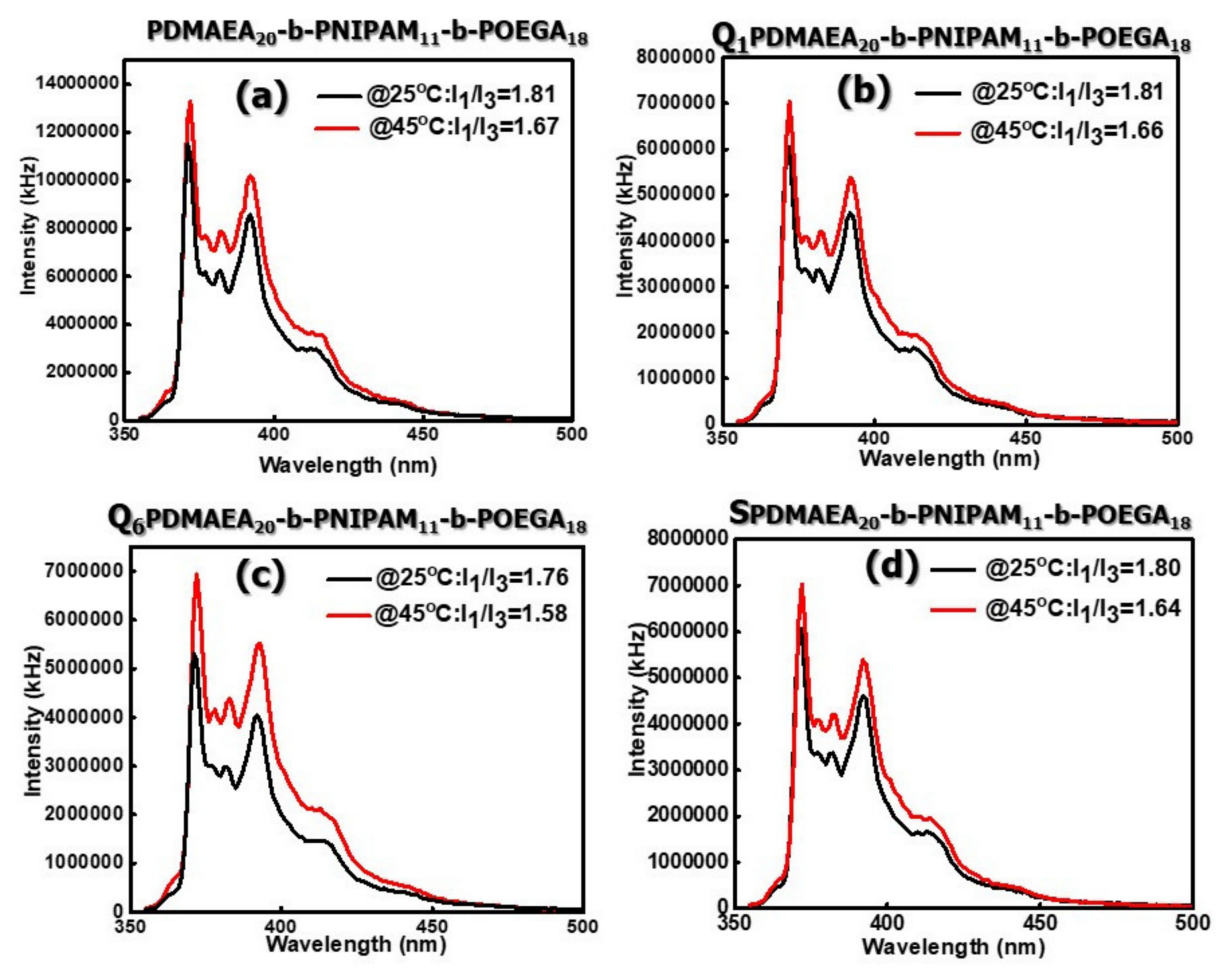
Fluorescence spectra for PDMAEA_20_-b-PNIPAM_11_-b-POEGA_18_ (**a**), Q_1_PDMAEA_20_-b-PNIPAM_11_-b-POEGA_18_ (**b**), Q_6_PDMAEA_20_-b-PNIPAM_11_-b-POEGA_18_ (**c**), and SPDMAEA_20_-b-PNIPAM_11_-b-POEGA_18_ (**d**) triblock terpolymers in aqueous solutions media at 25 °C and 45 °C.

In Figure 8, the fluorescence spectra for amine and quaternized triblock terpolymers are presented. For PDMAEA_20_-b-PNIPAM_11_-b-POEGA_18_, Q_1_PDMAEA_20_-b-PNIPAM_11_-b-POEGA_18_, and SPDMAEA_20_-b-PNIPAM_11_-b-POEGA_18_ terpolymers aggregates, the I_1_/I_3_ values are relatively high even at 45 °C (although lower compared to those at 25 °C). It is known that the PNIPAM does not display an absolutely hydrophobic character because of the existence of hydrogen bonding interactions with water molecules, even at temperatures above the LCST values. This feature of PNIPAM blocks in conjunction with the partially protonated PDMAEA block in the PDMAEA_20_-b-PNIPAM_11_-b-POEGA_18_ terpolymer, the fully protonated PDMAEA block in the Q_1_PDMAEA_20_-b-PNIPAM_11_-b-POEGA_18_ terpolymer, and the zwitterionic charges on the PDMAEA block in the SPDMAEA_20_-b-PNIPAM_11_-b-POEGA_18_ result in the relatively high I_1_/I_3_ values. However, for the Q_6_PDMAEA_20_-b-PNIPAM_11_-b-POEGA_18_ aggregates, the determined I_1_/I_3_ values at 45 °C are lower than that of aggregates at 25 °C, indicating the formation of less hydrophilic nanodomains detected by the pyrene within the aggregates. The presence of hydrophobic alkyl side chains on the PDMAEA block seems to affect the thermoresponsive behavior of the PNIPAM block and reduces the overall hydrophilicity of the system upon temperature rise above the LCST.

## 4. Conclusions

A dual-responsive PDMAEA_20_-b-PNIPAM_11_-b-POEGA_18_ triblock terpolymer was successfully synthesized via sequential RAFT polymerization. The tertiary amine groups attached to the PDMAEA block were chemically modified via quaternization and sulfobetainization reactions in order to produce Q_1_PDMAEA_20_-b-PNIPAM_11_-b-POEGA_18_ and Q_6_PDMAEA_20_-b-PNIPAM_11_-b-POEGA_18_ cationic polyelectrolytes utilizing methyl iodide and 1-iodohexane as the quaternizing agents and SPDMAEA_20_-b-PNIPAM_11_-b-POEGA_18_ zwitterionic polyampholyte utilizing 1,3-propanesultone as the surfobetainization agent. Molecular characterization results evidenced the synthesis of a triblock terpolymer with well-controlled molecular weights and close to targeted compositions, as well as the successful chemical modifications of the PDMAEA block. 

Self-assembly studies in aqueous solutions as a function of pH and temperature revealed that the amine-based triblock terpolymer displays thermoresponsive behavior, even though the amount of PNIPAM block is considerably lower than the other constituting blocks. As indicated by compiled DLS/SLS measurements, the amine triblock terpolymer self-assembles into nanostructures in solutions of different pH while the temperature increases above the LCST-like transition, where the temperature-responsive PNIPAM block should compose the inner part, and the pH-responsive PDMAEA and hydrophilic POEGA blocks should occupy the outer part of the self-assembled nanostructures. Moreover, light scattering results on all chemically modified triblock terpolymers showed evidence of the formation of nanoassembled structures by increasing the solution temperature above the LCST. The chemically modified triblock terpolymers self-assemble into larger aggregates in the whole temperature range compared to the amine functionalized triblock terpolymer as a result of electrostatic repulsions of the permanently charged quaternary amine groups of the modified PDMAEA block. The chemical functionalization reactions of the PDMAEA block affect the thermoresponsiveness of the PNIPAM block, causing the formation of spherical and more compact nanostructures upon temperature rise. The light scattering and FS results have shown that the carboxylate functional group placed at the end of the terpolymer chain, the variations in pH solution, the functionalization of the PDMAEA block and the overall macromolecular architecture of the synthesized triblock terpolymer have a significant effect on the temperature-responsive self-assembly of the terpolymers in aqueous solutions and on the structure of the formed aggregates. Based on the physicochemical data on the amine-based triblock terpolymer and its quaternized counterparts, especially their capability of self-assembling into aggregates in response to temperature variations, the terpolymers may be considered potential candidates as components of non-viral gene delivery and protein vectors or/and as thermoresponsive nanocarriers for the encapsulation of hydrophobic drugs.

## Figures and Tables

**Table 1:** Molecular characteristics of all synthesized triblock terpolymers.

SAMPLE	M_w_^a^ (×10^4^) (g/mol)	M_w_/M_n_^a^	%wt PDMAEA	%wt PNIPAM	%wt POEGA
**PDMAEA**	0.28	1.17	-		
**PDMAEA-b-PNIPAM**	0.41	1.28	69 ^b^	31 ^b^	
**PDMAEA_20_-b-PNIPAM_11_-b-POEGA_18_**	1.28	1.57	22 ^b^	10 ^b^	68 ^b^
**QPDMAEA_20_-b-PNIPAM_11_-b-POEGA_18_**	1.56 ^c^	-	36 ^c^	8 ^c^	55 ^c^
**Q_6_PDMAEA_20_-b-PNIPAM_11_-b-POEGA_18_**	1.70 ^c^	-	41 ^c^	8 ^c^	51 ^c^
**SPDMAEA_20_-b-PNIPAM_11_-b-POEGA_18_**	1.52 ^c^	-	34 ^c^	8 ^c^	58 ^c^

**^a^** Measured by SEC in THF containing 5% Et_3_N, M_w_ values refer to the apparent molecular weights since the SEC instrument is calibrated with PS standards. **^b^** Determined by ^1^H-NMR. **^c^** M_w_ and composition values are calculated assuming 100% conversion.

**Table 2:** Light scattering results for triblock amine terpolymers and their chemically modified analogues at neutral pH and at selected temperatures.

SAMPLE	T (°C)	M_w, app_ (×10^6^) (g/mol) ^a^	N_agg_^a^	R_h_ (nm) ^b^	ζ-Potential (mV) ^c^
**PDMAEA_20_-b-PNIPAM_11_-b-POEGA_18_**	25	0.3	23	26	+2.27
	45	4.2	328	73	+4.60
**Q_1_PDMAEA_20_-b-PNIPAM_11_-b-POEGA_18_**	25	0.15	9	126	+22.1
	45	1.8	115	105	+42.7
**Q_6_PDMAEA_20_-b-PNIPAM_11_-b-POEGA_18_**	25	0.8	47	169	+19.2
	45	7.3	429	137	+25.7
**SPDMAEA_20_-b-PNIPAM_11_-b-POEGA_18_**	25	0.4	26	98	+12.1
	45	5.2	342	160	+2.7

^a^ Determined by static light scattering (SLS), ^b^ Determined by dynamic light scattering (DLS, at an angle of 90°), ^c^ Determined by electrophoretic light scattering (ELS).

## Supplementary Materials

The following are available online at https://www.mdpi.com/2073-4360/12/6/1382/s1, Figure S1: Hydrodynamic radius distributions from Contin analysis for PDMAEA_20_-b-PNIPAM_11_-b-POEGA_18_ at acidic pH and at 25 °C, 45 °C, and 55 °C. Figure S2: Hydrodynamic radius distributions from Contin analysis for PDMAEA_20_-b-PNIPAM_11_-b-POEGA_18_ at acidic pH and at 25 °C, 45 °C, and 55 °C.
