# Supplementary material for: Effects of Chemical Modifications on the Thermoresponsive Behavior of a PDMAEA-b-PNIPAM-b-POEGA Triblock Terpolymer
Source: Polymers (Basel). 2020 Jun 19;12(6):1382. doi: 10.3390/polym12061382 (PMC7361810; doi:10.3390/polym12061382)
Supplement: Supplementary file 1 [file polymers-12-01382-s001.pdf]

polymers MDPI

Supplementary material

Effects of chemical modifications on the thermoresponsive behavior of a PDMAEA-b-PNIPAM-b-POEGA triblock terpolymer

Despoina Giaouzi[1], Stergios Pispas[1,*]

[1]Theoretical and Physical Chemistry Institute, National Hellenic Research Foundation, 48 Vassileos Constantinou Avenue, Athens 11635, Greece; dgiaouzi@gmail.com

* Correspondence: pispas@eie.gr; Tel.: +30-210-727-3824

Received: date; Accepted: date; Published: date

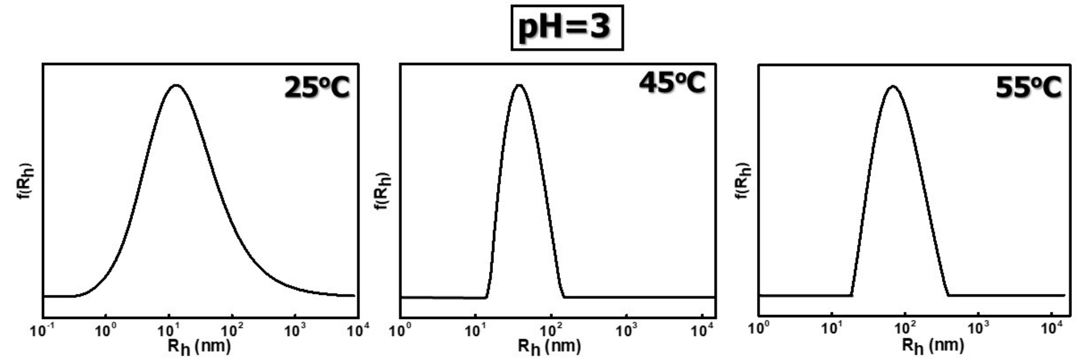

Figure S1. Hydrodynamic radius distributions from Contin analysis for $PDMAEA_{20}$-b-$PNIPAM_{11}$-b-$POEGA_{18}$ at acidic pH and at 25°C, 45°C, 55°C (DLS measurements were made at 90° angle).

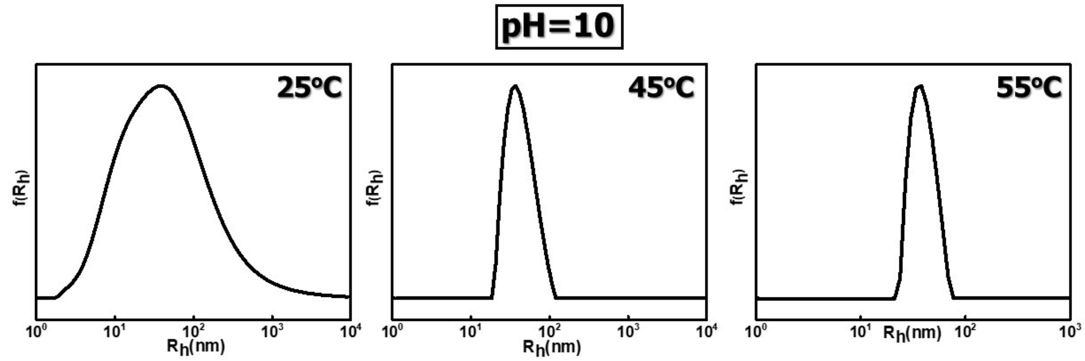

Figure S2. Hydrodynamic radius distributions from Contin analysis for $PDMAEA_{20}$-b-$PNIPAM_{11}$-b-$POEGA_{18}$ at acidic pH and at 25°C, 45°C, 55°C (DLS measurements were made at 90° angle).

© 2020 by the authors. Submitted for possible open access publication under the terms and conditions of the Creative Commons Attribution (CC BY) license (http://creativecommons.org/licenses/by/4.0/).
